# Mechanism underlying hippocampal long-term potentiation and depression based on competition between endocytosis and exocytosis of AMPA receptors

**DOI:** 10.1038/s41598-020-71528-3

**Published:** 2020-09-07

**Authors:** Tomonari Sumi, Kouji Harada

**Affiliations:** 1 Research Institute for Interdisciplinary Science, Okayama University, 3-1-1 Tsushima-Naka, Kita-ku, Okayama, 700-8530 Japan; 2 Department of Chemistry, Faculty of Science, Okayama University, 3-1-1 Tsushima-Naka, Kita-ku, Okayama, 700-8530 Japan; 3 Department of Computer Science and Engineering, Toyohashi University of Technology, 1-1 Hibarigaoka, Tempaku-cho, Toyohashi, Aichi, 441-8580 Japan

**Keywords:** Biophysical models, Long-term depression, Long-term potentiation

## Abstract

N-methyl-D-aspartate (NMDA) receptor-dependent long-term potentiation (LTP) and long-term depression (LTD) of signal transmission form neural circuits and thus are thought to underlie learning and memory. These mechanisms are mediated by AMPA receptor (AMPAR) trafficking in postsynaptic neurons. However, the regulatory mechanism of bidirectional plasticity at excitatory synapses remains unclear. We present a network model of AMPAR trafficking for adult hippocampal pyramidal neurons, which reproduces both LTP and LTD. We show that the induction of both LTP and LTD is regulated by the competition between exocytosis and endocytosis of AMPARs, which are mediated by the calcium-sensors synaptotagmin 1/7 (Syt1/7) and protein interacting with C-kinase 1 (PICK1), respectively. Our result indicates that recycling endosomes containing AMPAR are always ready for Syt1/7-dependent exocytosis of AMPAR at peri-synaptic/synaptic membranes. This is because molecular motor myosin V_b_ constitutively transports the recycling endosome toward the membrane in a Ca^2+^-independent manner.

## Introduction

**Figure 1:**
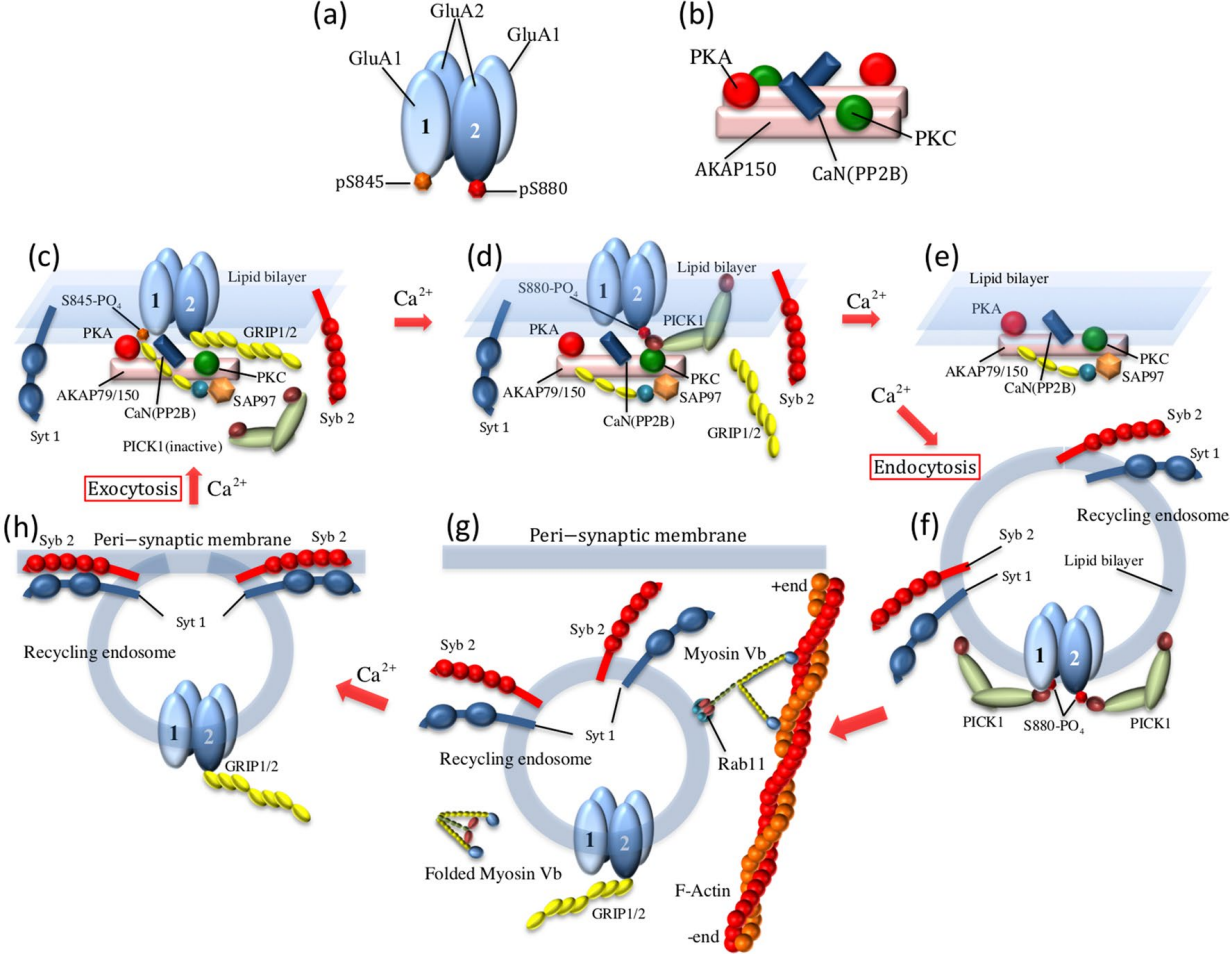
AMPAR trafficking model at hippocampal postsynaptic neurons. (**a**) AMPAR which consists of two GluA1 (GluR1) and two GluA2 (GluR2) subunits is the most predominant AMPAR subtype in hippocampal neurons^4^. The serine-845 site (S845) of GluA1 is phosphorylated and dephosphorylated by protein kinase A (PKA)^35^ and protein phosphatase 2B (PP2B, or Calcineurin, CaN)^36^, respectively. The serine-880 site (S880) of GluA2 is phosphorylated and dephosphorylated by protein kinase C (PKC)^37^ and protein phosphatase 2 (PP2A), respectively. (**b**) A-kinase anchoring protein 150 (AKAP150)^34,43,44^ is an anchoring protein that organizes PKA, PP2B, and PKC for phosphoregulation of AMPARs at the synaptic membrane, and thus acts as the AKAP signaling complex. The AKAP signaling complex forms the dimer as shown in (**b**) (though, for simplification, not shown in (**d**–**e**)). (**c**–**h**) Experimentally characterized elementary processes involved in the AMPAR trafficking cycle at a hippocampal postsynaptic neuron. (**c**) A model of tethering the GluA1 and GluA2 subunits to the AKAP150 signaling complex at the synaptic membrane through SAP97 and GRIP1, respectively^34,43,44^. (**d**) A model of phosphorylation and dephosphorylation reactions of the AMPAR due to Ca^2+^ signaling. Dephosphorylation of the S845 site of GluA1 is caused by PP2B^36^, and SAP97 dissociates from GluA1. The S880 site of GluA2 is phosphorylated by PKC, and PICK1 binds to the GluA2 subunit instead of GRIP1^37,38^. (**e**,**f**) An endocytic model for synaptic vesicles containing AMPAR mediated by the calcium-sensor PICK1^37,38,47^. (**g**) Active transport of the recycling endosomes by molecular motor myosin V_b_^40–42^. In the recycling endosome, PP2A causes the dephosphorylation of S880 of GluA2, and GRIP1 instead of PICK1 binds to GluA2^46^. (**h**) An exocytic model of the recycling endosome triggered by Ca^2+^-sensor synaptic vesicle protein synaptotagmin 1 (Syt1) together with synaptotagmin 7 (not shown) and synaptobrevin-2 (Syb2)/VAMP2, complexin (not shown), amongst others^22,23,48–50,61^.

Synaptic plasticity is generally regulated by the release of various neurotransmitters from the presynaptic membrane and/or by varying the density, types, and properties of neurotransmitter receptors at the postsynaptic membrane. NMDA (N-methyl-D-aspartate) receptor-dependent long-term potentiation (LTP) and long-term depression (LTD) of signal transmission in excitatory neurons, such as hippocampal pyramidal neurons, is thought to underlie the formation of neuronal circuits during learning and memory^1–3^. Fast excitatory neurotransmission in the mammalian brain is predominantly mediated by the AMPA (α-amino-3-hydroxy-5-methyl-4-isoxazolepropionic acid) receptor (AMPAR) at the postsynaptic membrane. AMPAR is tetrameric ion channel composed of the subunits GluA1–A4 (or named as GluR1–R4). In hippocampal pyramidal neurons, the GluA1/A2 heterotetramer (Fig. 1a) is the most dominant AMPAR subtype, followed by the GluA2/A3 heterotetramer^4^. It is well established that AMPAR trafficking in postsynaptic neurons plays a decisive role in the induction of LTP and LTD^5–8^, whereas the dominant pathway and regulatory mechanism for AMPAR trafficking remains a controversial issue.

NMDA receptor-dependent LTP and LTD are triggered by standard high-frequency stimulations (e.g., one or more trains of 100 Hz stimulation)^9,10^ and low-frequency stimulations (LFS; e.g., 700–900 pulses at 1 Hz)^11–14^, respectively. It is widely accepted that a high rise and lower rise in intracellular Ca^2+^ concentrations mediated by NMDA receptor activation are required for the expression of LTP and LTD, respectively. Nevertheless, it has been reported that a channel blocker for Ca^2+^ influx through the NMDA receptor does not block LTD in the hippocampal neurons of immature rodents, thereby suggesting that LTD is attributed to an ion-channel-independent, metabotropic form of NMDA-receptor signaling^15^. Subsequently, it was carefully reexamined how varying extracellular Ca^2+^ levels and blocking NMDA-receptor channel ion flux with the same channel blocker affected the induction of LTD. It was reconfirmed that the LTD induced by a standard LFS in hippocampal neurons of both adult and immature rodents was dependent on ionotropic NMDA-receptor signaling^16^.

For more than 20 years, it has been known that Ca^2+^/calmodulin-dependent protein kinase (CaMKII) activity is necessary for LTP. Ca^2+^ ions incorporate into the cell through NMDA receptor and bind to calmodulin (CaM). Consequently Ca^2+^/CaM is bound to CaMKII. If CaMKII is knocked out, Ca^2+^/CaM binds directly to NMDA receptors, resulting in inactivation of the NMDA receptor channel and an accordingly significantly lower rise in Ca^2+^ concentrations^17,18^. On the other hand, if Ca^2+^/CaM-bound CaMKII binds to the NMDA receptor and inhibits the direct binding of Ca^2+^/CaM to the NMDA receptor, this maintains the channel activity of the NMDA receptor. This is one of the most important CaMKII functions at the early stage of NMDA receptor-dependent LTP induction process. In addition, the Ca^2+^/CaM-CaMKII bound to the NMDA receptor phosphorylates GluA1^19^, resulting in increased channel conductance of AMPAR^20^. The activation of AMPAR by CaMKII also plays a crucial role in the induction of LTP. In the present study, we build a network model to reproduce NMDA-receptor dependent bidirectional synaptic plasticity of hippocampal neurons. All of the molecular mechanisms incorporated into the model work downstream of these CaMKII functions; thus, the normal activities of CaMKII are necessary and implicitly assumed in our network model. In fact, NMDA receptor-dependent Ca^2+^-influx is introduced in the network model as the input for LTP and LTD simulations. In addition, it is assumed that the strength of LTP and LTD induction is in proportion to the change in the AMPAR population on the postsynaptic membrane. Thus, the effects of GluA2-lacking Ca^2+^-permeable AMPARs are not taken into consideration in our simulations. However, this assumption does not influence the prediction of LTP expression by our network model, because it has been demonstrated that LTP in the hippocampal CA1 region does not require insertion or activation of GluA2-lacking AMPARs^21^.

NMDA-receptor dependent LTP is thought to occur by incorporating AMPARs into the synaptic membrane through either the exocytosis of the recycling endosome at peri-synaptic/synaptic membranes^22–25^, the cell surface long-range lateral diffusion of AMPAR from the dendrites/extrasynaptic region^26–29^, or a combination of the two. Recently, Penn et al. examined the effects of crosslinking immobilization of pre-existing membrane AMPARs on early LTP and observed slow continuous development of early LTP without short-term potentiation (STP)^26^. They also observed that blocking exocytosis of AMPARs completely abolished early LTP and caused only STP induction^26^. The former and latter respectively indicate the following: (1) lateral diffusion movement of AMPARs exocytosed at the synaptic/peri-synaptic membranes as well as the extrasynaptic membranes is significantly suppressed due to obstacle effects arising from jamming/crowding of crosslinked AMPARs, as predicted by computer simulations^30^; (2) although the recruitment of pre-existing membrane AMPARs from the peri-synaptic membrane works for the induction of STP, it is insufficient for maintaining the expression of early LTP. Therefore, the diffusion dynamics on the membrane strongly affect the form of LTP expression and play a crucial role in the rapid short-range diffusion relocation of AMPARs from the peri-synaptic to the synaptic membrane. It was also demonstrated by the duration of STP^26^ that the short-range diffusion relocation takes, at most, only 2 min. On the other hand, the observed delay of LTP induction caused by the obstacle effects provides no definitive evidence that the long-range lateral diffusion pathway of AMPARs from the dendrites/ extrasynaptic region is the predominant pathway for early LTP than the short-range diffusional relocation pathway of exocytic AMPARs from the peri-synaptic membranes. Indeed, exocytosis of GluA1, not only in dendrites but also in synaptic spines including synaptic/peri-synaptic membranes, has been demonstrated using the SEP-GluA1 imaging technique, which resolved the controversy^27,31^. Alternatively, that observation does not necessarily exclude the short-range diffusional relocation pathway from the promising AMPAR recruitment pathway during LTP. This is because the impaired diffusional movement of AMPARs exocytosed at the peri-synaptic membranes, which is caused by the obstacle effects due to jamming/crowding of crosslinked AMPARs, can yield a delay to early LTP. This hypothesis suggests that the total amount of AMPARs exocytosed at the peri-synaptic membranes should be sufficiently greater than that at the synaptic membrane due to the larger area of peri-synaptic membranes.

In general, the normal lateral diffusion process is isotropic; thus, the flux of lateral diffusion from a place in the extrasynaptic region toward the synaptic membrane and from this place in the opposing direction are in equilibrium (for instance^32^). Therefore, the cell surface lateral diffusion movement is thought to be ineffective for long-range directional transport of AMPARs. Furthermore, the Ca^2+^-dependent/independent biased surface diffusion mechanism of AMPAR toward the synaptic membrane from the dendrites/extrasynaptic region remains unclear. The long-range transport of AMPARs should necessitate directional non-equilibrium active movement, e.g., driven by molecular motors that consume ATP as fuel (for instance^33^).

## Method

### Network model

In the network model presented here, we incorporate elemental processes involved in AMPAR trafficking cycles that have been characterized experimentally for hippocampal postsynaptic neurons (Fig. 1). The AMPAR trafficking cycle is constructed around the phosphorylation/dephosphorylation dynamics of AMPAR at the synaptic membrane (Fig. 1c,d)^34–36^, the endocytosis of vesicles containing AMPAR (Fig. 1e,f)^37,38^, the transport of the recycling endosomes^8,39^ by molecular motor myosin V_b_^40–42^ (Fig. 1g), and exocytosis of the recycling endosomes^22,23^, followed by the incorporation of AMPARs into the peri-synaptic/synaptic membranes (Fig. 1h). The key facilitators of the AMPAR trafficking cycle^43^ are summarized as follows: the GluA1/A2 heterotetramer (Fig. 1a); A-kinase anchoring protein 150 (AKAP150)^34,44,45^, which dimerizes (as shown in Fig. 1b but not shown in Fig. 1c–e) and forms a signaling complex along with protein kinase A (PKA)^35^, protein kinase C (PKC)^34^, and protein phosphatase 2B (PP2B, also known as Calcineurin, CaN)^36^ (Fig. 1b); the AMPAR interacting proteins such as synaptic associated protein 97 kDa (SAP97)^43,44^ and glutamate receptor interacting protein 1/2 (GRIP1/2)^43,46^; and two calcium-sensor proteins: protein interacting with C-kinase 1 (PICK1)^37,38,47^ and synaptotagmin 1/7 (Syt1/7)^22,23,48–50^.

The GluA1/A2 heterotetramer becomes localized during *diffusional relocation* at the synaptic membrane by tethering to AKAP150 via SAP97, which binds to the serine-845 site (S845) of GluA1^44,45,51,52^, and via GRIP1, which binds to the dephosphorylated serine-880 site (S880) of GluA2^46^, (if S845 of GluA1 has been phosphorylated by cAMP-dependent PKA^35,53^, Fig. 1c). This is because the AMPAR interacting proteins SAP97 and GRIP1 have PDZ domains^43^ and thus bind preferentially to AKAP150, which also has PDZ domains^44^. If S845 of GluA1 is dephosphorylated by Ca^2+^-dependent PP2B^36^, SAP97 dissociates from GluA1 (Fig. 1d). Furthermore, phosphorylation of the S880 GluA2 site by Ca^2+^-dependent PKC^37,54^ causes dissociation of GRIP1, at which point PICK1, which also has PDZ domains, binds to GluA2 instead^37^ (Fig. 1d). Increases in Ca^2+^ concentrations through NMDA receptors induces the active state of the Ca^2+^-sensor PICK1 and then the endocytosis of AMPARs, which are comprised of two GluA1s with dephosphorylated S845 and two GluA2s with phosphorylated S880^37,38,47,55^. Consequently, the endocytic vesicles containing these AMPARs undergo diffusion in the cytosol (Fig. 1e,f). At this point, it is assumed that the S880 of GluA2s in recycling endosomes are dephosphorylated by PP2A ^56,57^, PICK1 dissociates from GluA2, whilst GRIP1 binds to GluA2 (Fig. 1g) ^43,46^. It has been demonstrated using GRIP1 and/or GRIP2 knock-out mice that GRIP1 and GRIP2 regulate AMPAR trafficking ^46^. However, for simplification, we assume that the functions of GRIP2 are also taken into consideration through the model of GRIP1 in the present study.

We explicitly take into consideration the active transport of recycling endosomes containing AMPARs by myosin V_b_ in our network model. Myosin V_b_ binds to the recycling endosome via Rab11 and transports it toward the peri-synaptic/synaptic membrane ^31,40–42^ (Fig. 1g). Although Ca^2+^-dependent activity of myosin V has been reported ^58^, the run speed and mean run length are almost constant at the Ca^2+^ concentrations that we consider in this study ^59^. Therefore, we model the myosin-V_b_ transport of recycling endosomes as a Ca^2+^-independent constitutive movement toward the peri-synaptic/synaptic membrane, which is driven by ATP hydrolysis energy. Exocytosis of the recycling endosomes transported by myosin V_b_ is mediated at the peri-synaptic/synaptic membrane by the Ca^2+^-sensor synaptic vesicle protein synaptotagmin 1 (Syt1), together with synaptotagmin 7 (Syt7) (not shown in Fig. 1), synaptobrevin-2/VAMP2, and complexin (not shown in Fig. 1), amongst others^22,23,49,50,60,61^. As a result, the AMPARs are incorporated into the peri-synaptic/synaptic membrane (Fig. 1h). In fact, exocytosis of GluA1 not only in dendrites but also in synaptic spines including peri-synaptic/synaptic membranes has been demonstrated using the SEP-GluA1 imaging technique^31^. The localization of Syt1 at the peri-synaptic/synaptic membranes, which has been observed for hippocampal postsynaptic neurons^48^, also supports the Syt1-mediated exocytosis pathway of the recycling endosomes. The exocytic AMPARs incorporated into the peri-synaptic membrane are relocated into the synaptic membrane via a local diffusional movement, which takes, at most, only 2 min, as shown by the observation on the duration of STP^26^. In the present study, we did not introduce enough fine space resolution into our network model to identify whether AMPARs are located at the peri-synaptic or synaptic membrane. However, the ~ 2-min delay of AMPAR incorporation into the synaptic membrane after exocytosis at the peri-synaptic membrane would be taken into consideration via effective rates for synaptotagmin-dependent exocytosis. In addition, the diffusional relocation dynamics of AMPARs at the synaptic membrane plays a crucial role in meeting and interacting with the signaling complex AKAP150, thereby resulting in the stabilization of AMPARs at the synaptic membrane by tethering to AKAP150 via SAP97 and GRIP1, as mentioned above.

**Figure 2:**
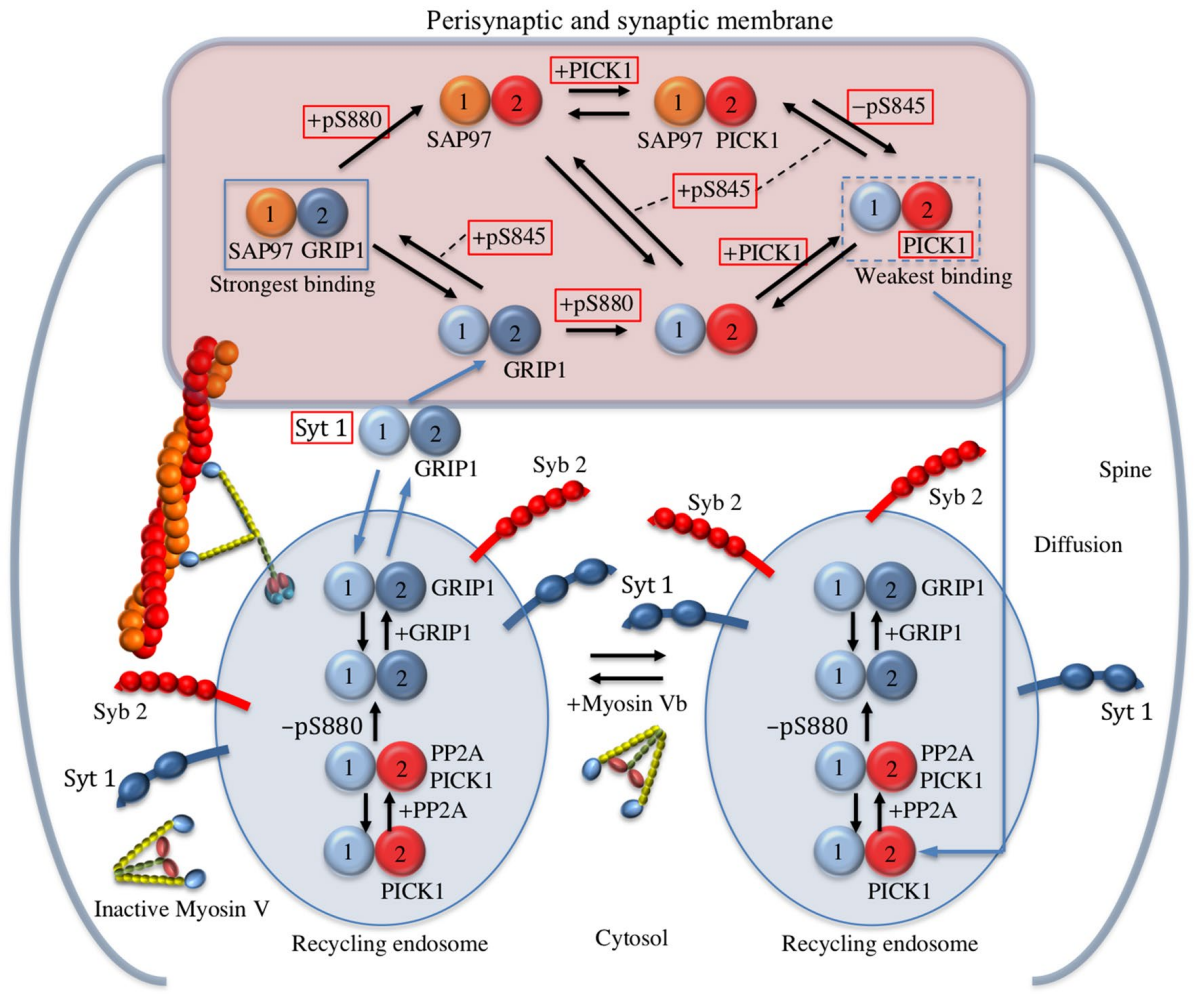
The network model of AMPAR trafficking that mediates hippocampal LTP and LTD. For simplification, the AMPAR is schematically depicted as two particles corresponding to the GluA1 and GluA2 subunits. This network model is based on the experimental observations that are summarized in Fig. 1. Here, the reaction network on the phosphorylation/dephosphorylation dynamics of GluA1 and GluA2 is also displayed schematically. The recycling endosomes containing AMPAR are actively transported by myosin V_b_ toward the peri-synaptic/synaptic membrane. The lateral diffusion relocation of AMPAR is assumed to occur during the phosphorylation and dephosphorylation of AMPAR at the synaptic membrane, in addition to the local diffusional relocation movement of the exocytic AMPAR from the peri-synaptic to synaptic membrane. The phosphorylation state of GluA1 and GluA2 regulates localization of the AMPARs at the synaptic membrane via interactions with various AMPAR interacting proteins (SAP97, GRIP1, PICK1)^43^.

As a result, we propose the network model for bidirectional hippocampal synaptic plasticity that consists of 285 reaction equations for 191 components, including: (1) Ca^2+^ dynamics, (2) the phosphorylation/dephosphorylation dynamics of the tetrameric AMPAR ion channel subtype GluA1/A2, (3) the endocytosis/exocytosis dynamics of the AMPARs, mediated respectively by the Ca^2+^-sensors PICK1 and Syt1, and (4) the recycling endosome active transport by molecular motor myosin V_b_ toward the peri-synaptic/synaptic membrane (Fig. 2, Supplementary Table S2). Thus, our network model involves the previously proposed model on bidirectional synaptic plasticity based on the phosphorylation/dephosphorylation of GluA1 S845^62^. The validity of the network model is also demonstrated by reproducing the impairments of LTP and LTD caused respectively by genetic chemical inhibition of myosin V_b_ transport^42^ and by AKAP150ΔPIX knock-in mice, which selectively disrupt the anchoring of PP2B to AKAP150^36^. The network model reveals that the competition between exocytosis caused by Syt1, and endocytosis caused by PICK1 depends on transient increases in intracellular Ca^2+^ concentrations, which therefore regulate AMPAR trafficking resulting in LTP or LTD. The obtained results also indicate that the constitutive active transport of the recycling endosomes by myosin V_b_ increases the basal concentration of the recycling endosomes localized on the peri-synaptic/synaptic membrane surface, so that the AMPARs, which are ready for Syt1-mediated exocytosis, can be immediately incorporated into the postsynaptic membranes by LTP stimulation. This insertion mechanism resolves the long-standing contradiction between the prompt LTP induction and the several-minute delay on the starting time of myosin V_b_ transport following LTP^42^.

### Simulations

We built the network model for hippocampal LTP and LTD which is consisted of a well-mixed single compartment. The ordinary differential equations for the network model comprised of 285 reaction equations on 191 components were solved using COPASI biochemical system simulator (ver. 4.23)^63^. The detailed description of the model including components, reactions, and parameters can be found in the SI text. We employed the steady-state concentrations as the initial condition and performed the simulations of the LTP and LTD inductions for 5,400 s.

## Results

### Simulated Ca^2+^ pulses induce LTP and LTD

**Figure 3:**
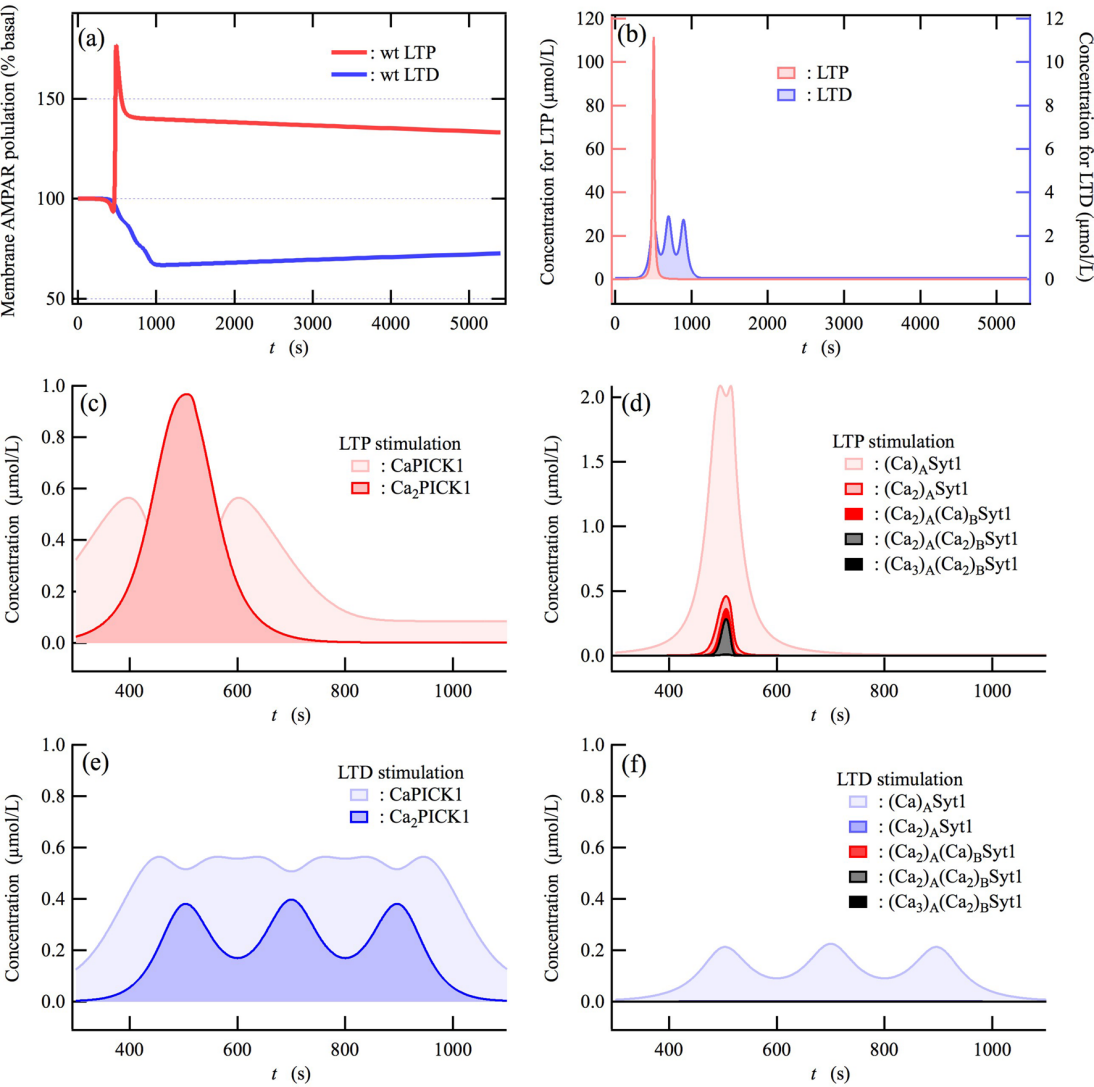
Hippocampal LTP and LTD are regulated by the activation of Ca^2+^-sensors Syt1 and PICK1 in response to Ca^2+^ influx. (**a**) Time course of the membrane AMPAR population, indicating induction of LTP and LTD. Here, 100% represents the basal AMPAR population at the membrane. (**b**) Ca^2+^-pulse concentrations corresponding to the LTP and LTD stimulation are shown on the left and right axis, respectively. (**c**–**f**) The concentrations of Ca^2+^-binding species of PICK1 (**c,e**) and Syt1 (**d**,**f**) as a function of time (*t*) during LTP (c and d) and LTD induction (**e**,**f**). In (**f**), the concentration of multiple Ca^2+^-binding species other than (Ca)_A_Syt1 is too small to see at this scale, indicating that Syt1 is mostly not activated. Therefore, Ca^2+^-dependent exocytosis mediated by Syt1 occurs during LTD.

The magnitudes of LTP and LTD depend on experimental protocols, though typical forms of LTP and LTD have been found. For instance, it has been observed that high-frequency stimulation (HFS) at 100 Hz for 1 s sharply induces LTP, increasing synaptic transmission up to ~ 200% compared to basal levels^9,36,64^. The magnitude drops rapidly to ~ 150%, and then slowly decreases toward basal transmission level. Our network model reproduces the change in the membrane AMPAR level that is qualitatively consistent with experimentally observed LTP induction (Fig. 3a), when we mimic a rapid transient increase in intracellular Ca^2+^ concentration caused by HFS-induced Ca^2+^ influx through NMDA receptors, which is the input for LTP simulation, using a single Gaussian function (Fig. 3b). On the other hand, it has been found experimentally that induction of LTD requires LFS (700–900 pulses at 1 Hz)^11–14^. Synaptic transmission gradually decreases down to ~ 60%^11,13^, and then slowly increases toward the basal transmission level^12,14^. When the transient rise of intracellular Ca^2+^ caused by LFS is modelled using three Gaussian functions (Fig. 3b), our network model reproduces the experimentally observed membrane AMPAR level for LTD^11^ (Fig. 3a). In addition, we found that the network model reproduces LTP and LTD inductions under a wide range of Ca^2+^ peak pulse amplitudes (see Supplementary Fig. S1). Furthermore, we confirmed that these LTP and LTD inductions were held even if we used two sigmoid functions to mimic Ca^2+^ influx instead of the Gaussian functions (see Supplementary Fig. S2). These results indicate the validity of the network model for bidirectional synaptic plasticity. Here, it is noted that the network model reproduces the slow reduction in the amplitude of LTP and LTD toward the basal condition, which has been observed experimentally. Thus, the LTP yielded by the network model, which is based on AMPAR trafficking, corresponds to the early phases of LTP. The relaxation of LTP and LTD is mediated by constitutive endocytosis and exocytosis of AMPAR, thus indicating that the constitutive flux of the recycling endosome occurs under basal conditions.

### PICK1 is activated by both LTP and LTD stimulation whereas synaptotagmin 1 is mainly activated by LTP stimulation

In our network model, the Ca^2+^-sensors Syt1 and PICK1 play a dominant role in the exocytosis and endocytosis of AMPARs that regulate AMPAR trafficking during LTP and LTD induction. We built biochemical models of Syt1 and PICK1 based on experimentally determined association constants of Ca^2+^ ions to Syt1^60^ and PICK1^55^ (see the SI text). Syt1 has two Ca^2+^-binding domains, C2A and C2B, which bind three and two Ca^2+^ ions and directly interact with the recycling endosome and plasma membrane, respectively^50^. PICK1 has two Ca^2+^-binding domains, one each on the N-terminus and C-terminus, which interact with the phosphorylated S880 of GluA2 and the synaptic plasma membrane, respectively^37,38^. It has been demonstrated that Ca^2+^-binding site mutations of Syt1 in both the C2A and C2B domains block hippocampal LTP^22^. Therefore, it would be appropriate to use Ca^2+^-binding constants of Syt1 to model a Ca^2+^-dependent regulating factor on the exocytosis mediated by Syt1 together with Syt7, synaptobrevin-2/VAMP2, and complexin, amongst others^22,23,49,50,60,61^. During LTP stimulation, the concentrations of multiple Ca^2+^-binding species of Syt1, (Ca_2_)_A_Syt1, (Ca_2_)_A_(Ca)_B_Syt1, and (Ca_2_)_A_(Ca_2_)_B_Syt1, rapidly rise in relation to the increase in [(Ca)_A_Syt1]. Here and hereafter, we use square brackets to refer to concentrations. However, at this scale, [(Ca_3_)_A_(Ca_2_)_B_Syt1] does not show a significant increase (Fig. 3d).[Ca_2_PICK1] increases with a delay following the transient increase in [CaPICK1] (Fig. 3c). Therefore, both Syt1 and PICK1 are activated by LTP stimulation. Nevertheless, LTP is induced because the Syt1-mediated exocytosis overcomes the PICK1-mediated endocytosis. In contrast, at this scale during the LTD stimulation, increases in the concentration of multiple Ca^2+^-binding species of Syt1 are not seen (Fig. 3f), whereas [Ca_2_PICK1] and [CaPICK1] rise in relation to the rise in [Ca^2+^] (Fig. 3e). As a result, the PICK1-mediated endocytosis overcomes the Syt1-mediated exocytosis, causing the induction of LTD (the numerical results will be provided below). The difference in Ca^2+^-dependent activity of PICK1 and Syt1 during the LTP and LTD stimulation is attributable to that in Ca^2+^-binding affinity of these proteins (see Supplementary Fig. S3). Graupner and Brunel proposed that the total times when Ca^2+^ transient spends above depression and potentiation thresholds of synaptic transmission determined LTD and LTP induction^65^. The former and latter might correspond to Ca^2+^ concentrations that activate PICK1-dependent endocytosis and Syt1-dependent exocytosis in our network model.

It has been reported that the regulatory mechanism of AMPAR trafficking on hippocampal synaptic plasticity of rodents is development dependent. In fact, LTD is modestly affected in juvenile PICK1-knock out (KO) mice, whereas LTD induced by LFS is obviously reduced in PICK1-KO adult mice^66^. Although many factors other than PICK1 work in Ca^2+^-dependent endocytosis of AMPAR during LTD especially in hippocampal neurons of juvenile rodents, PICK1 plays a dominant role in the regulation of Ca^2+^-dependent endocytosis in adult rodents together with other proteins including clathrin and dynamin^67^. Thus, the regulatory mechanism of hippocampal bidirectional synaptic plasticity presented in this study is valid, especially for adult rodents. Very recently, it was demonstrated using synaptotagmin 3 (Syt3) KO that Syt3 is actually involved in the *endocytosis* of AMPARs during LTD^68^. However, the Ca^2+^ affinity of Syt3 has been reported as being tenfold higher than that of Syt1^69^, at levels more similar to PICK1. Therefore, Syt3-mediated endocytosis does not prevent the Ca^2+^-dependent regulatory mechanisms of exocytosis and endocytosis presented here, and the network model would reproduce the bidirectional synaptic plasticity even if it was also taken into consideration.

### Competition between exocytosis and endocytosis during the induction of LTP and LTD

**Figure 4:**
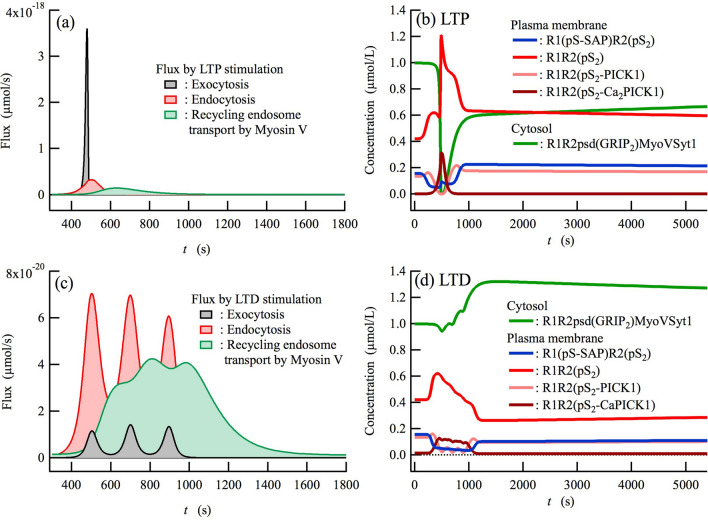
Competition between exocytosis and endocytosis of AMPARs yields LTP and LTD. (**a**,**c**) Total excess fluxes of exocytosis, endocytosis, and myosin V_b_ transport of recycling endosomes during (**a**) LTP stimulation and (**c**) LTD stimulation. (**b**,**d**) The time course of concentrations for predominant components during (**b**) LTP and (**d**) LTD.

As predicted from the Ca^2+^-dependent activation of Syt1 and PICK1 (Fig. 3c–f), LTP stimulation causes exocytic and endocytic fluxes simultaneously, whereas the exocytic flux should be much larger than the endocytic flux (Fig. 4a). On the other hand, the endocytic flux should be much larger than the exocytic flux during LTD stimulation (Fig. 4c). Unexpectedly, we find that the maximum endocytic flux in response to LTD stimulation (~ 7 × 10^–20^ µmol/s, Fig. 4c) is considerably smaller than the maximum endocytic flux in response to LTP stimulation (~ 32 × 10^–20^ µmol/s, Fig. 4a). Indeed, the total endocytosis of AMPARs induced by LTD stimulation (2.5 × 10^–17^ µmol) is smaller than that induced by the LTP stimulation (4.1 × 10^–17^ µmol). This is because the Ca^2+^ concentration during the LTP stimulation is significantly higher than during LTD. Nevertheless, LTD is induced by a smaller endocytic flux than during LTP stimulation because during LTD, the exocytic flux is sufficiently smaller than the endocytic flux. Likewise, LTP is induced even though the endocytic flux is larger than that following LTD stimulation because during LTP stimulation the exocytic flux is sufficiently higher than the endocytic flux. It is remarkable that an increase in the level of AMPAR internalized by the endosome after LTP induction could be observed experimentally^70^. In addition, it has been observed that NMDA-receptor-dependent LTP and LTD are impaired in PICK1-KO mice where PICK1-dependent endocytosis is inhibited^66,71^. These observations and our simulation results showing an impairment of both the LTP and LTD (see Supplementary Fig. S4) are the convincing evidence that supports our competition mechanism of bidirectional synaptic plasticity.

### Endocytic vesicles newly generated during LTP and LTD are transported by myosin V_b_ toward the peri-synaptic membrane

Endocytic vesicles containing AMPARs newly generated during the induction of LTP and LTD undergo diffusion in the cytosol as a recycling endosome. During the diffusion movement these recycling endosomes bind to molecular motor myosin V_b_ via Rab11 and are actively transported by it toward the peri-synaptic/synaptic membrane^40–42^. The long-term flux by myosin V_b_ transport is found in Fig. 4a,c. This indicates that the myosin V_b_ transport of recycling endosomes continues for a while after LTP and LTD are induced. Indeed, such active transport continues for ~ 7 and ~ 13 min from the onset of the LTP and LTD stimulation, respectively (Fig. 4a,c). The result obtained for LTP is consistent with experimental observations where the cooperative movements of recycling endosomes and myosin V_b_ molecules start a few minutes later than the LTP induction, and continue for several minutes^42^.

### Recycling endosomes transported by myosin V_b_ are localized on the surface of the peri-synaptic membrane, and thus are already prepared for exocytosis

We assume that the myosin V_b_ active transport takes place in a Ca^2+^-independent manner based on experimental observations^58,59^. Thus, the recycling endosomes in the cytosol are constitutively transported by myosin V_b_ toward the peri-synaptic and synaptic membranes. Consequently, the recycling endosomes are expected to become localized on the membrane surface under basal conditions. In fact, we see that the most dominant components in the cytosol under basal conditions (*t* = 0 s) are pre-exocytic recycling endosomes bound to the peri-synaptic/synaptic membrane surface, namely, R1R2psd(GRIP_2_)MyoVSyt1 (Fig. 4b,d). Such localization is necessary for the immediate exocytosis mediated by Syt1 after LTP stimulation. Specifically, this causes the rapid incorporation of AMPARs into the peri-synaptic/synaptic membranes during LTP induction. Sharp decreases in [R1R2psd(GRIP_2_)MyoVSyt1] occur immediately after LTP stimulation until it is depleted by the Syt1-mediated exocytosis. Simultaneously, the AMPAR population at the membranes increases due to Syt1-mediated exocytosis, then immediately begins to decrease after it reaches its peak (see Fig. 3a). Such a rapid decrease in the AMPAR population following the prompt increase is interpreted as being due to PICK1-mediated endocytosis following Syt1-mediated exocytosis, as seen in the excess fluxes during LTP (Fig. 4a). The increase in [R1R2psd(GRIP_2_)MyoVSyt1] following depletion (Fig. 4b) is therefore attributed to the myosin V_b_-transport of the endosomes newly internalized into the cytosol through endocytosis (Fig. 4a). Likewise, endocytic vesicles internalized during LTD induction are also transported by myosin V_b_ and are present in greater quantities on the membrane surface than under basal conditions (Fig. 4d), thus playing an important role in exocytosis due to subsequent LTP stimulation.

## Discussion

### Assessment of the validity of the network model for hippocampal LTP and LTD

To confirm the validity of the network model for bidirectional synaptic plasticity, we applied the model to two experimental observations: (1) reduction in LTP induction through genetic chemical inhibition of myosin V_b_ transport^42^, and (2) impaired LTD induction in AKAP150ΔPIX knock-in mice where the anchoring of PP2B to AKAP150 is selectively disrupted^36^. The former is useful to reveal how myosin V_b_ transport affects LTP induction. This is not a trivial issue, because the myosin V_b_ transport starts a few minutes later than the LTP induction, and the movement of recycling endosomes continues for ~ 10 min^42^. The latter is important to reveal how the competition between the phosphorylation/dephosphorylation of GluA1 S845 by PKA and PP2B affects LTD induction. Indeed, on the basis of localization of AMPARs at the synaptic membrane regulated by phosphorylation of GluA1 S845, a model of bidirectional synaptic plasticity has been proposed^62^.

**Figure 5:**
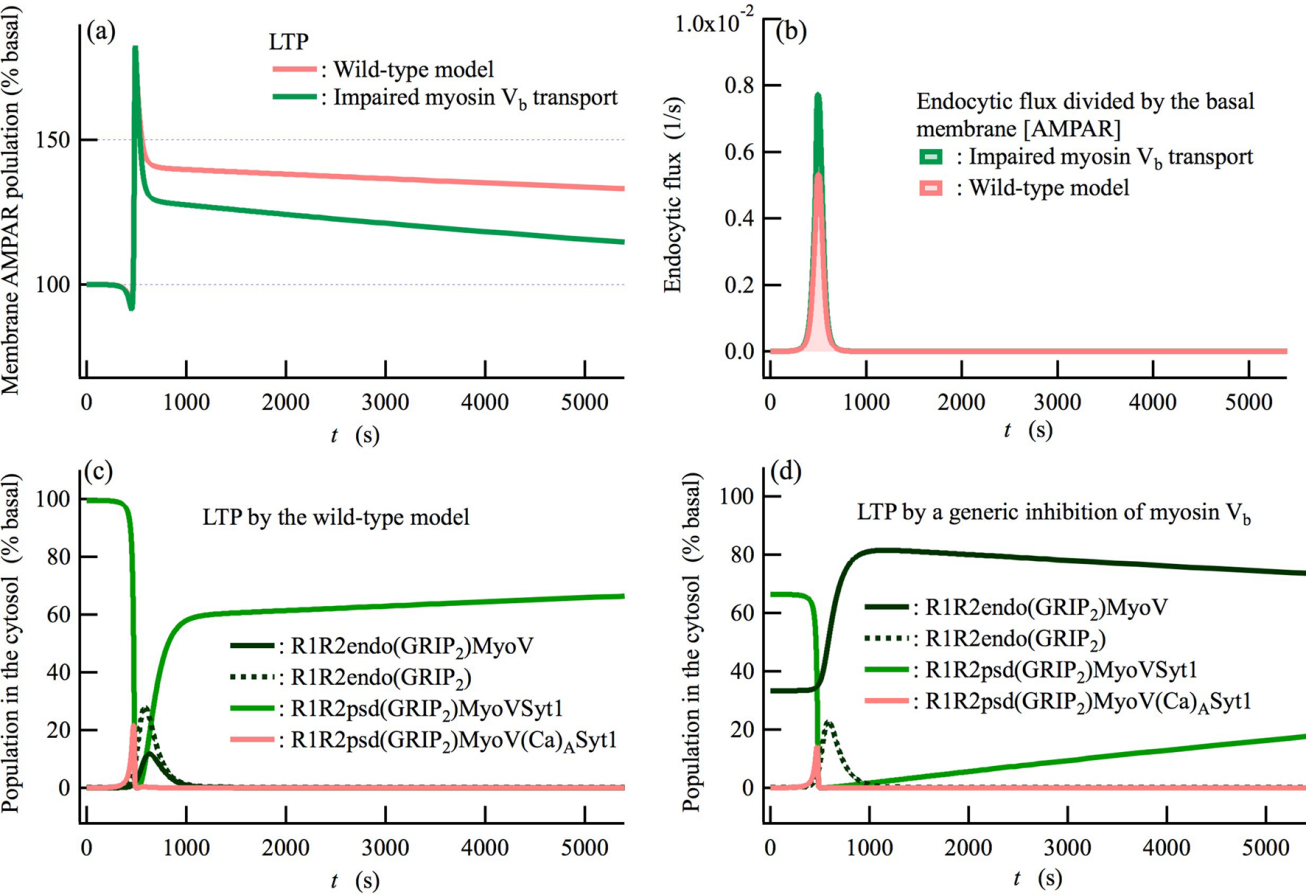
Myosin V_b_ transport predominantly governs the long-term behavior of LTP induction. (**a**) Time courses of the membrane AMPAR population obtained for the wild-type and genetically inhibited model of myosin V_b_ transport^42^. (**b**) Normalized endocytic fluxes defined as the endocytic flux divided by the basal [AMPAR] at the membrane. (**c**,**d**) The populations of predominant components in the cytosol during LTP induction for (**c**) the wild-type model and (**d**) a model with inhibited myosin V_b_ transport, shown as functions of time (*t*). Myosin V_b_-binding recycling endosome in the cytosol, [R1R2endo(GRIP_2_)MyoV], is increased under basal conditions by the inhibition of myosin V_b_ transport, and furthermore is increased during impaired LTP induction, as seen in (**d**).

**Figure 6:**
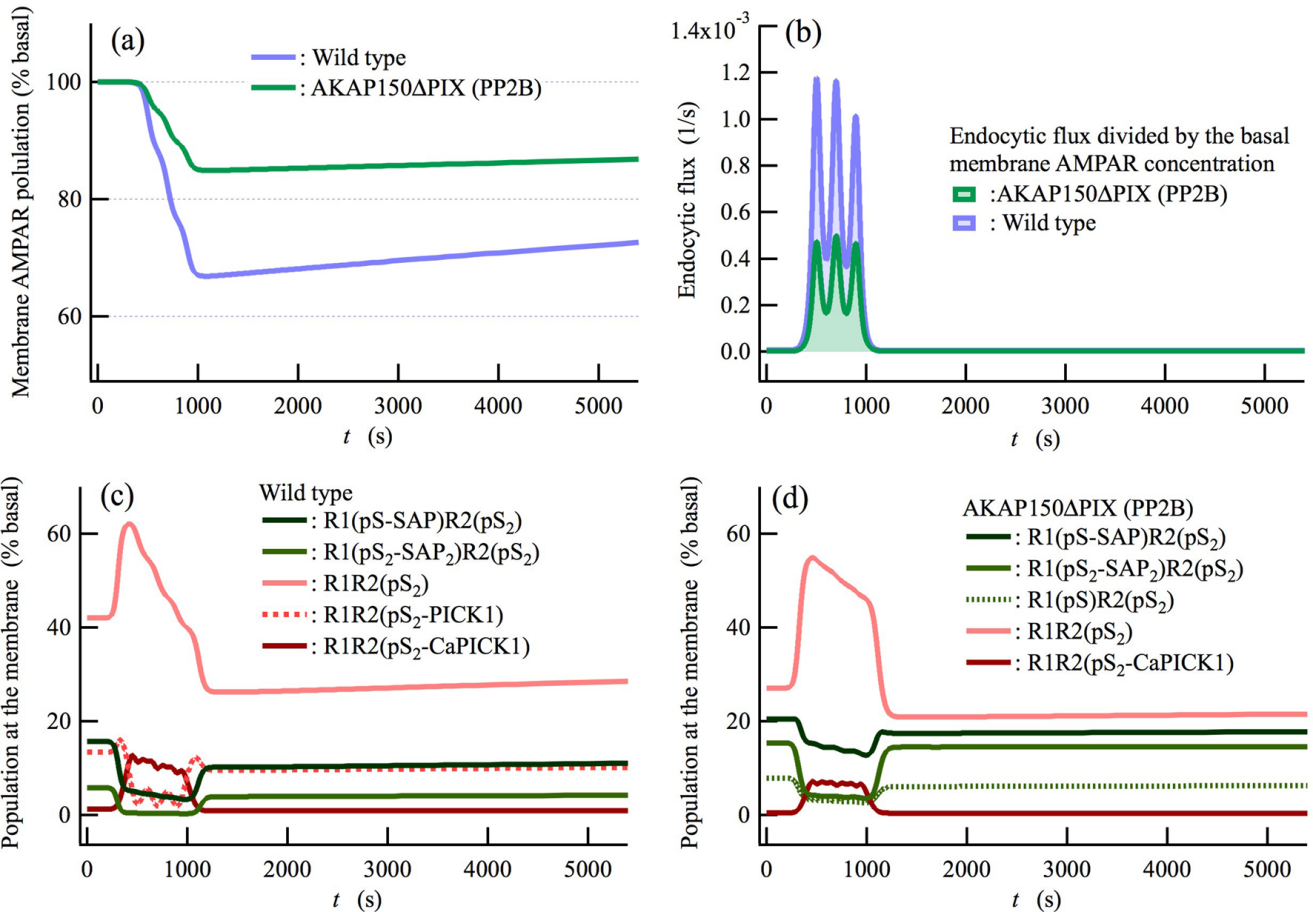
Dephosphorylation of GluA1 S845 by protein phosphatase 2B (PP2B, Calcineurin) regulates the induction of LTD. (**a**) Time courses of the membrane AMPAR population for wild-type and PP2B-anchoring deficient AKAP150ΔPIX mice^36^. (**b**) Normalized endocytic fluxes, defined as the endocytic flux divided by the basal [AMPAR] at the membrane. (**c**,**d**) The populations of the main (top five) components at the membrane during induction of LTD for (**c**) the wild-type model and (**d**) the AKAP150ΔPIX model are shown as functions of time (*t*). PICK1-binding AMPARs at the membrane, which have been prepared for PICK1-mediated endocytosis, such as R1R2(pS2-PICK1) and R1R2(pS2-CaPICK1) (**c**), are decreased by disrupting the PP2B-dependent dephosphorylation of GluA1 S845. It is noted that R1R2(pS_2_-PICK1) shown in (**c**) is not displayed in (**d**) because it is present at levels lower than the other components shown here. On the other hand, the AMPARs that are bound to the membrane through tethering of GluA1 to AKAP150 via SAP97, such as R1(pS-SAP)R2(pS_2_) and R1(pS_2_-SAP_2_)R2(pS_2_), are increased by the inhibition of the PP2B-dependent dephosphorylation of GluA1 S845.

Our network model reproduces the experimentally observed reduction in LTP due to the inhibition of myosin V_b_ transport (Fig. 5a)^42^, i.e., that the LTP magnitude is lower in the early stage (~ *t* = 700 s) than in the wild-type model, and that the following reduction of LTP toward basal levels is accelerated. The model also reproduces experimentally observed LTD impairment by disrupting dephosphorylation of GluA1 S845 by PP2B (Fig. 6a)^36^. Additional demonstrations of the network model are presented in Supplementary Fig. S5. These simulation results are also useful to further support the validity of the bidirectional regulatory mechanism of the network model on the induction of both LTP and LTD.

### The accelerated reduction of LTP toward basal levels reflects inhibition of myosin V_b_ transport

Inhibition of myosin V_b_ transport does not affect the rapid increase in the membrane AMPAR population just after the onset of LTP stimulation (Fig. 5a). However, following this rapid increase, the AMPAR population steeply drops down to lower levels than that of the wild-type model (~ *t* = 700 s), showing an impairment of LTP induction. The steep decrease after the initial increase can be attributed to PICK1-mediated endocytosis, which occurs concurrently during LTP induction (Fig. 4a). Normalized endocytic fluxes, defined as the total endocytic flux divided by the basal [AMPAR] at the membrane, indicate that the ratio of endocytic AMPARs relative to the total amount of membrane AMPARs is increased by the inhibition of myosin V_b_ transport (Fig. 5b). Whilst the increase in the normalized endocytic flux is unexpected, it can be interpreted through changes in the population that are related to PICK1-bound species, to which myosin V_b_ does not directly bind (Supplementary Fig. S6). These observations show that the reduction in LTP in the early stage (~ *t* = 700 s) is attributable to *indirect* effects of the inhibition of myosin V_b_ transport. In fact, the cooperative movement of recycling endosomes and myosin V_b_ molecules starts several minutes later than LTP induction.

On the other hand, the accelerated reduction in LTP magnitude toward basal levels compared with the wild-type model can be interpreted as the *direct* effects due to inhibition of myosin V_b_ transport. Indeed, the concentration of recycling endosomes to which myosin V_b_ binds, [R1R2endo(GRIP_2_)MyoV], is significantly increased due to the inhibition of myosin V_b_ transport not only under basal conditions (*t* = 0 s) but also at times after the induction of LTP (e.g., *t* = 1,000 s, Fig. 5d). In contrast, in the wild-type model, [R1R2endo(GRIP_2_)MyoV] slightly increases only during LTP stimulation, and immediately vanishes due to myosin V_b_ transport (Fig. 5c). The slow increase in the concentration of the recycling endosomes on the membrane surface, [R1R2psd(GRIP_2_)MyoV], by inhibiting myosin V_b_ transport reflects the rate-limiting process in the constitutive cycle of AMPAR trafficking, which results in a faster reduction in LTP toward basal levels^42^.

### Phosphorylation/dephosphorylation dynamics of AMPARs at the synaptic membrane regulate AMPAR trafficking

AKAP150^34,44,45^ forms a signaling complex with PKA, PKC, and PP2B (Calcineurin, CaN, Fig. 1b) and affects the phosphorylation/dephosphorylation dynamics of the tetrameric AMPAR ion channel GluA1/A2 (Fig. 1c,d). The S845 site of GluA1 is phosphorylated and dephosphorylated by PKA and PP2B, respectively. The enzymes PKA and PP2B control the localization of GluA1/A2 to the synaptic membrane by tethering GluA1 to AKAP150 via SAP97, due to the fact that SAP97 preferentially binds to the phosphorylated S845 site of GluA1^35,36^. However, it remains unclear how these opposing effects of PKA and PP2B are regulated during the induction of LTD. To address this issue, AKAP150ΔPIX knock-in mice, where the anchoring of PP2B to AKAP150 is disrupted, have been developed. Using the knock-in mice, it has been demonstrated that inhibition of the interaction between AKAP150 and PP2B impairs hippocampal LTD induction^36^. In this study, we modeled hippocampal synapses for AKAP150ΔPIX knock-in mice by suppressing the dephosphorylation rate of GluA1 S845 by PP2B (Supplementary Table S5). The LTD induction is impaired in the AKAP150ΔPIX model compared with the wild-type model (Fig. 6a). In Fig. 6c,d, R1R2(pS_2_) species that bind SAP97, R1(pS-SAP)R2(pS_2_), and R1(pS_2_-SAP_2_)R2(pS_2_), show larger populations than species that do not bind SAP97 by disrupting PP2B-anchoring to AKAP150. This is because dephosphorylation of GluA1 S845 is suppressed in the AKAP150ΔPIX model. Consequently, the population of the main species in preparation for endocytosis, such as R1R2(pS_2_-PICK1) and R1R2(pS_2_-CaPICK1), is reduced in the AKAP150ΔPIX model, resulting in a decrease in the normalized endocytic flux (Fig. 6b). Note that R1R2(pS_2_-PICK1), which is displayed in Fig. 6c, is not shown in Fig. 6d owing to its lower levels than that of other species. These observations indicate that the competition between the phosphorylation/dephosphorylation of AMPARs at the synaptic plasma membrane affect AMPAR trafficking. In addition to the results shown here, we find that the activation levels of PKA and PP2B are not effectively regulated by differences in Ca^2+^ concentration during LTP and LTD stimulation (Supplementary Fig. S7). Thus, although the unbinding of GluA1 from the synaptic membrane is necessary for the LTD induction, the previous model based on only the GluA1-binding to /GluA1-unbinding from the synaptic membrane^62^ is insufficient to explain the NMDA receptor-dependent synaptic plasticity. The Ca^2+^-dependent competition between Syt1-mediated exocytosis and PICK1-regulated endocytosis is necessary as the dominant mechanism on LTD as well as LTP.

### Dynamics of recycling endosomes during LTP induction and the role of myosin V_b_ transport

**Figure 7:**
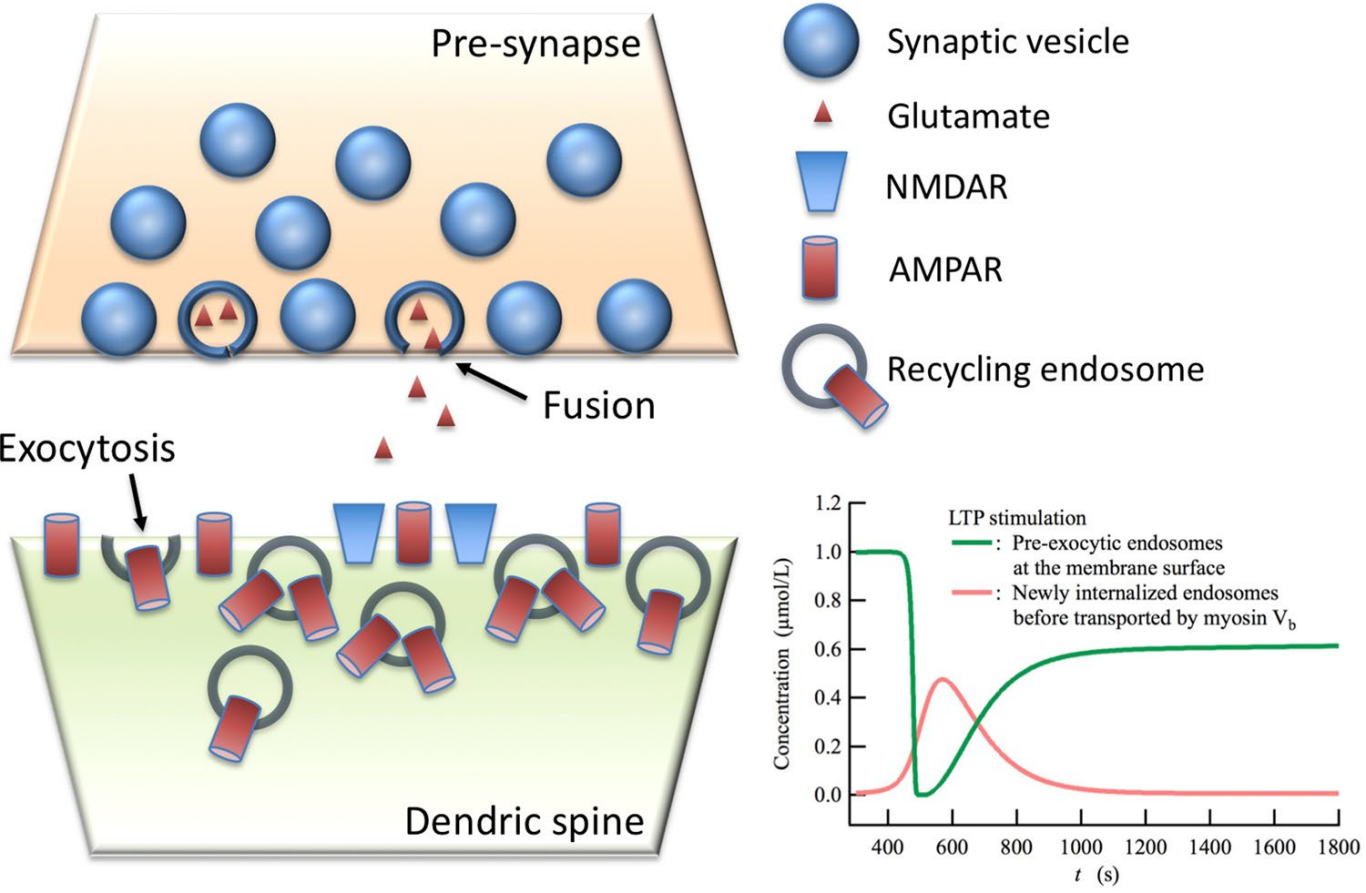
Schematic model of a hippocampal postsynaptic membrane. Recycling endosomes are localized on peri-synaptic/synaptic membrane surface under basal conditions, thus they are already prepared for Ca^2+^-dependent Syt1-mediated exocytosis resulting in the prompt incorporation of AMPARs into the membranes. The graph shows the time courses of the total concentrations for cytoplasmic components, namely, pre-exocytic recycling endosomes that are localized on the membrane surface and newly internalized endosomes, during LTP induction. The pre-exocytic recycling endosomes are steeply decreased by Syt1-mediated exocytosis, then are gradually increased by the myosin-V_b_ active transport of newly internalized recycling endosomes by PICK1-mediated endocytosis.

Myosin V_b_ is widely expressed in most neurons, including those in the hippocampus, which implies the possibility that myosin V_b_ could mediate endosomal trafficking during LTP induction^40,42,72^. Indeed, it has been demonstrated that the genetic chemical inhibition of myosin V_b_ motility through the binding of nonhydrolyzable PE-ADP impairs LTP induction^42^. On the other hand, on the basis of several experimental observations, it has been suggested that the cell surface long-range lateral diffusion pathway of AMPARs from the dendrites/extrasynaptic region to the synaptic membrane is the dominant mechanism for AMPAR recruitment during LTP^26–29^. Thus, it is deduced that during hippocampal LTP, AMPARs are recruited into the synaptic membrane through either Ca^2+^-dependent exocytosis at peri-synaptic/synaptic membrane, the cell surface long-range lateral diffusion movement, or a combination of the two. However, in spite of extensively observing numerous experimental evidences^22–25,31,73^, the Ca^2+^-dependent exocytosis pathway of the recycling endosome assisted by myosin V_b_ transport seems to have lately fallen out of favor as the main pathway for AMPAR trafficking. In fact, recently proposed models for cerebellar LTP and LTD have taken into account the lateral diffusion of AMPARs from the extrasynaptic area to the synaptic membrane as the main pathway of AMPAR trafficking^57,74^. In addition to the influential arguments that support the long-range lateral diffusion pathway^26–29^, one of the reasons for this in the case of hippocampal LTP arises from the following observation: LTP induction is immediately caused by the prompt incorporation of AMPARs into the synaptic membrane, whilst myosin-V_b_ transport starts several-minutes later than the onset of LTP stimulation, and continues for ~ 10 min^42^. These facts, that seem to contradict each other at first glance, are actually not problematic, at least in the context of hippocampal LTP. In our simulation, the flux from myosin V_b_ transport of newly internalized endosomes reaches a maximum at 2 min after the onset of LTP stimulation (Fig. 4a), and continues for a total of ~ 10 min (see the increase in [R1R2psd(GRIP_2_)MyoVSyt1] in Fig. 5c). Even so, LTP induction can still be reproduced by the network model. Such an apparent discrepancy can be resolved as follows: the prompt incorporation of AMPARs into the synaptic/peri-synaptic membranes followed by the rapid diffusional relocation of AMPARs from the peri-synaptic to synaptic membrane, which is required for the immediate induction of hippocampal LTP, is achieved by the Syt1-mediated exocytosis of recycling endosomes localizing on the membrane surface (Fig. 7). This is because the recycling endosomes have already been transported into the synaptic/peri-synaptic membrane surface by myosin V_b_ in a Ca^2+^-independent manner. This theoretical prediction is supported by the colocalization of Syt1 with endosomal vesicles^50^ and the localization of Syt1 at the peri-synaptic/synaptic membranes^48^. Taking the experimental observations discussed in this study and our simulation results together, we reach a plausible hypothesis that reconciles the long-standing controversy around the AMPAR trafficking pathway: the short-range diffusional relocation pathway of AMPARs exocytosed by Ca^2+^-dependent Syt1 at the peri-synaptic membranes can primarily contribute to AMPAR recruitment during LTP more than the cell surface long-range lateral diffusion pathway for AMPARs exocytosed at the dendrites/extrasynaptic region. In addition, to conciliate this hypothesis with Penn’s observations^26^, we can conclude that the total amount of AMPARs exocytosed at the peri-synaptic membranes should be sufficiently larger than that at the synaptic membrane due to the larger area of peri-synaptic membranes. Therefore, the short-range diffusional relocation pathway of exocytic AMPAR from the peri-synaptic membranes should be more dominant than the direct exocytosis pathway of AMPAR into the synaptic membrane.

## Supplementary information

Supplementary Information.
