# Supplementary material for: Mechanism underlying hippocampal long-term potentiation and depression based on competition between endocytosis and exocytosis of AMPA receptors
Source: Sci Rep. 2020 Sep 7;10:14711. doi: 10.1038/s41598-020-71528-3 (PMC7477194; doi:10.1038/s41598-020-71528-3)
Supplement: Supplementary file 1 — Supplementary Information. [file 41598_2020_71528_MOESM1_ESM.docx]

**Supplementary Information**

**Mechanism underlying hippocampal long-term potentiation and depression based on competition between endocytosis and exocytosis of AMPA receptors**

**Tomonari Sumi**^1,2^ **and Kouji Harada**^3^

^1^Research Institute for Interdisciplinary Science, Okayama University, 3-1-1 Tsushima-Naka, Kita-ku, Okayama 700-8530, Japan

^2^Department of Chemistry, Faculty of Science, Okayama University, 3-1-1 Tsushima-Naka, Kita-ku, Okayama 700-8530, Japan

^3^Department of Computer Science and Engineering, Toyohashi University of Technology, 1-1 Hibarigaoka, Tempaku-cho, Toyohashi, Aichi, 441-8580, Japan

**SUPPLEMENTARY INFORMATION FIGURES**

**
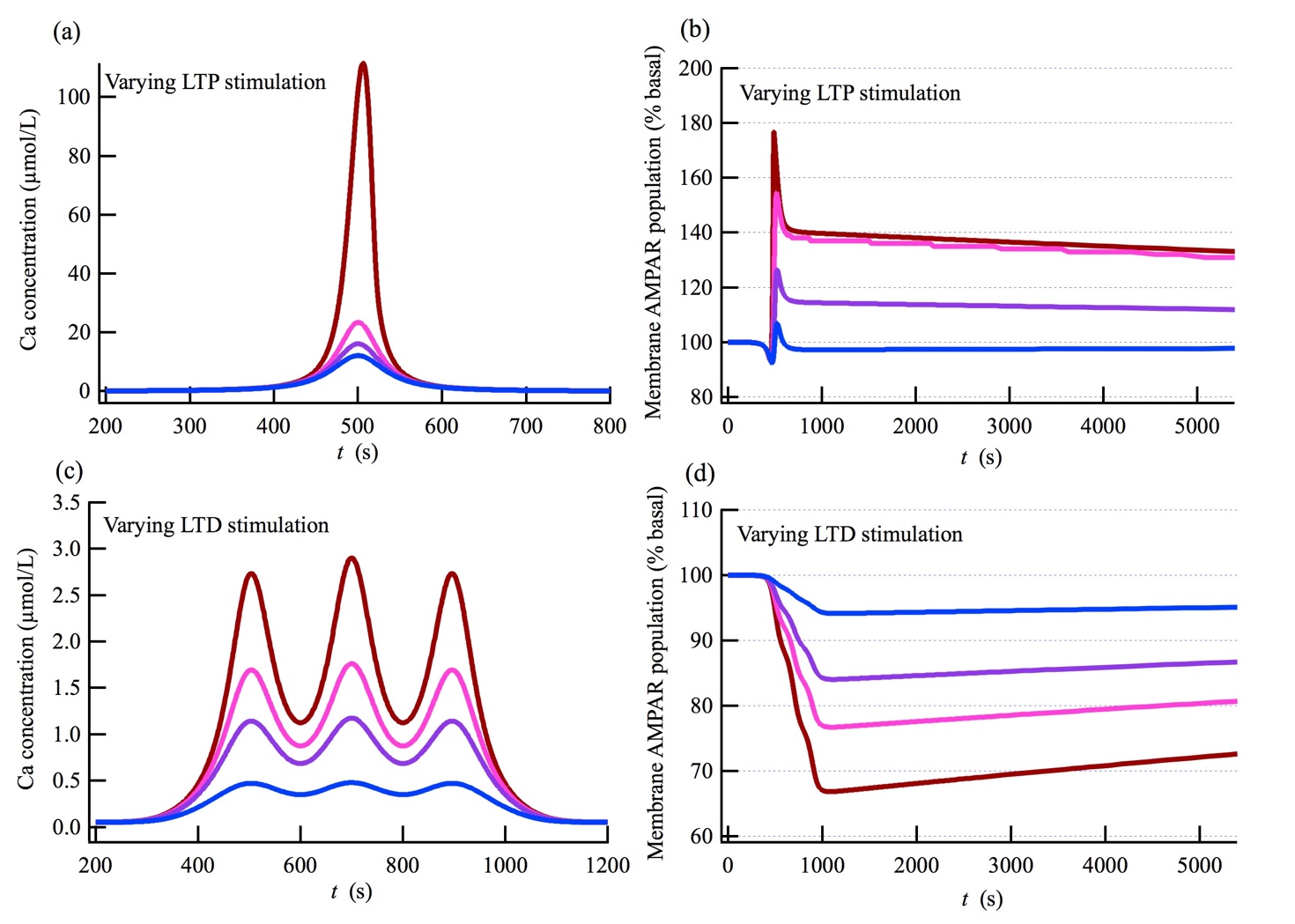
**

**Figure S1.** **Ca^2+^ peak pulse amplitude-dependent LTP and LTD inductions.** (a) The weaker the Ca^2+^ peak pulse amplitude is, (b) the weaker the LTP expression becomes. The Ca^2+^ pulses with each color in (a) induce the LTP expressions with corresponding color in (b). (c) The weaker the C^a2+^ peak pulse amplitudes are, (d) the weaker the LTD expression becomes. The trains of Ca^2+^ pulse with each color in (c) induce the LTD expressions with corresponding color in (d). The Ca^2+^ pulses with the strongest peak amplitude in (a) and (d) respectively correspond to Ca^2+^ pulses of the LTP and LTD stimulation shown in Fig. 3b.


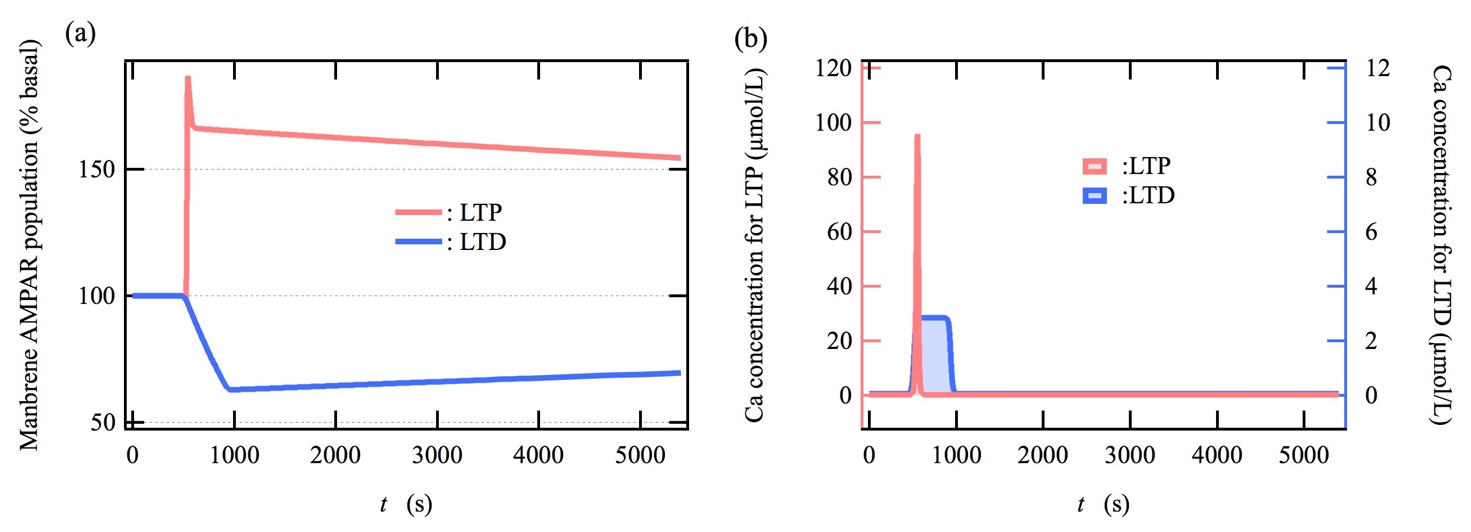


**Figure S2. LTP and LTD induction by using a two-sigmoid function model as the input Ca^2+^ influx instead of the Gaussian function model.** Time course of (a) AMPAR population and (b) Ca^2+^ concentration for LTP and LTD induction. The two-sigmoid function model used here for the LTP and LTD stimulation is explained in **SUPPLEMENTARY INFORMATION METHOD** (shown below).

**
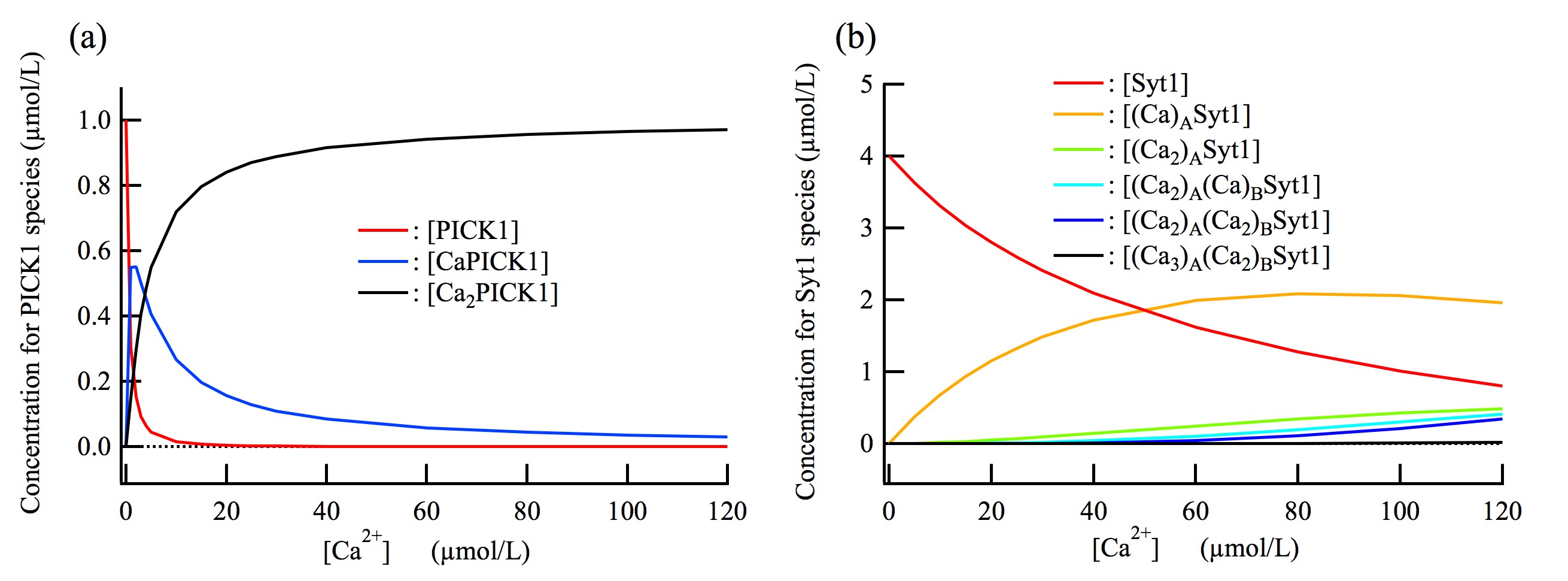
**

**Figure S3. Concentration variation of Ca^2+^-binding species of Syt1 and PICK1 as a function of Ca^2+^ concentration.**

**
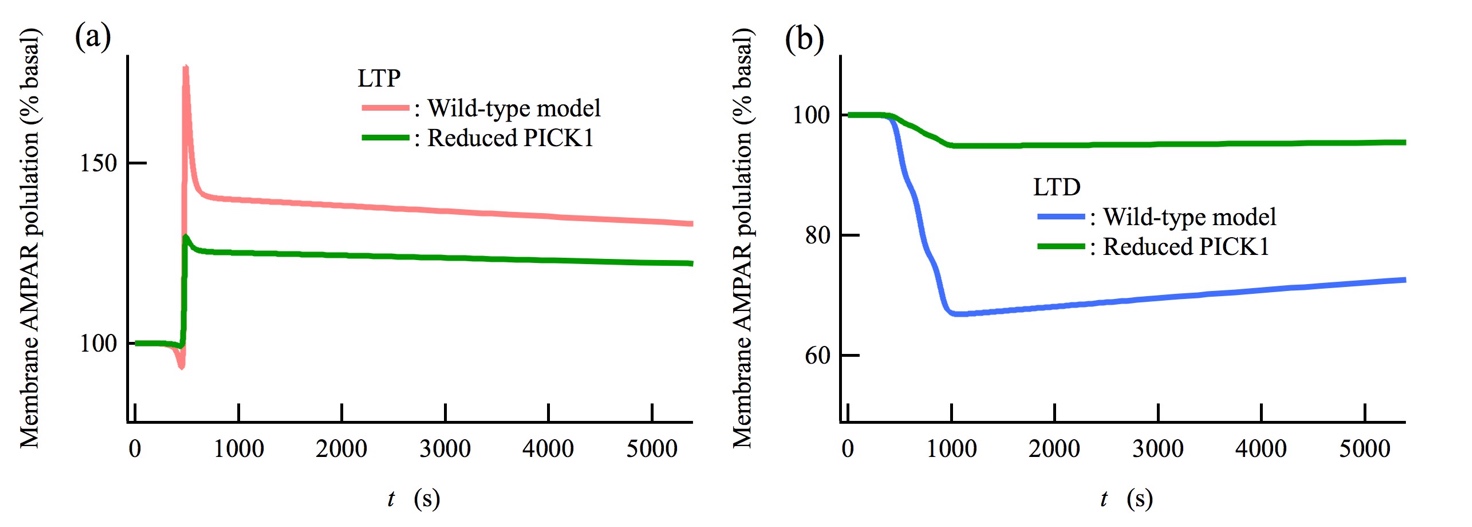
**

**Figure S4.** **A reduction of PICK1-dependent endocytosis impairs both the LTP and LTD inductions.** (a) LTP induced by the HFS. (b) LTD by the LFS. These results are comparable with the impairment of LTP and LTD inductions observed in PICK1-KO mice [1].


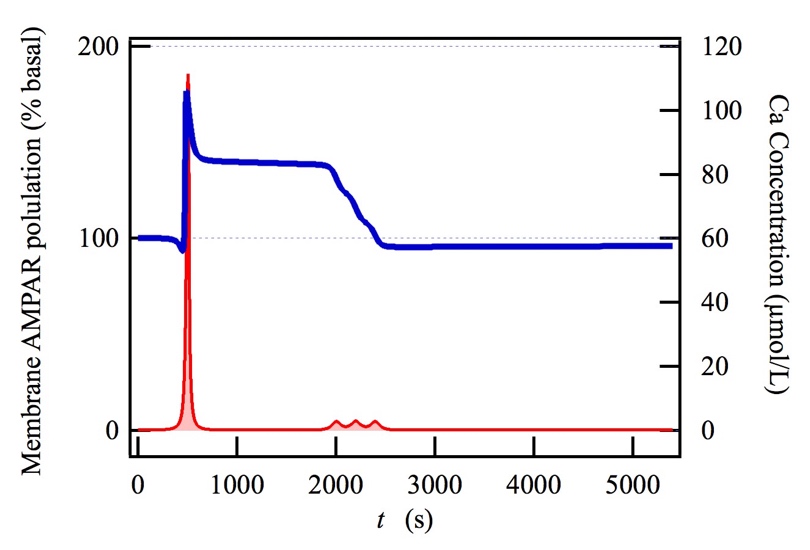


**Figure S5.** **The induction of LTP is depotentiated by LTD stimulation.** Time courses of (left axis) AMPAR population at the membrane and (right axis) Ca^2+^ concentration. The LTP and LTD stimulation are the same as used in Fig. 3 whereas the timing of LTD stimulation is delayed by 1500 s. The obtained time course is qualitatively consistent with experimentally observed one [2].

**
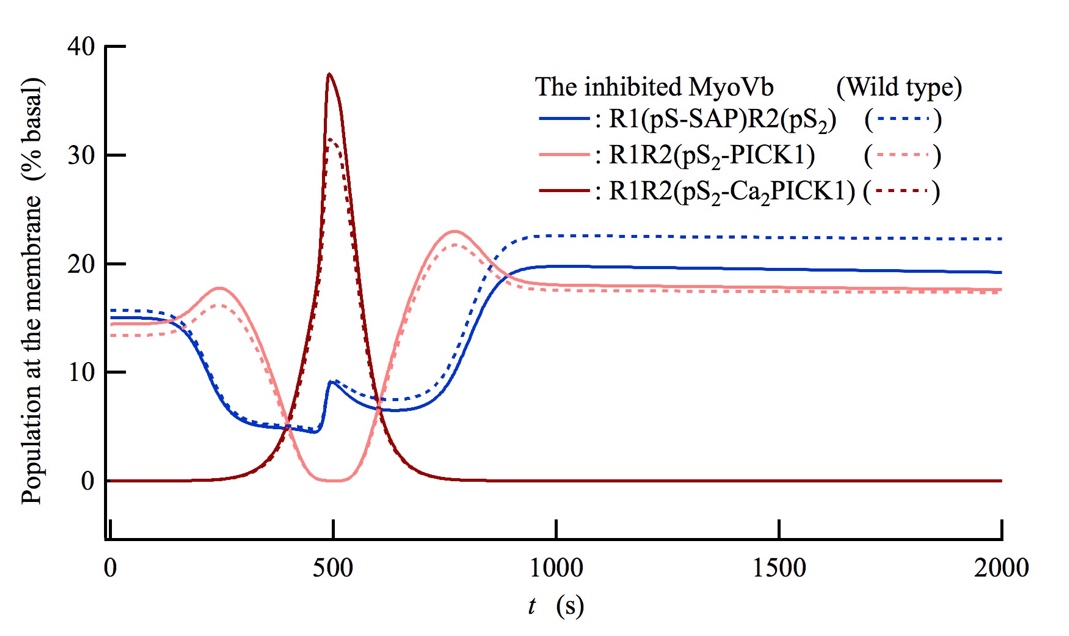
**

**Figure S6.** **An inhibition of myosin V_b_ transport affects the population of each component at the membrane.** The increase in the ratio of the amount of PICK-mediated endocytic AMPARs to the total amount of AMPARs at the membrane (Fig. 5b) can be interpreted on the basis of the slight increase in the population of PICK1-bound AMPARs as observed here.

**
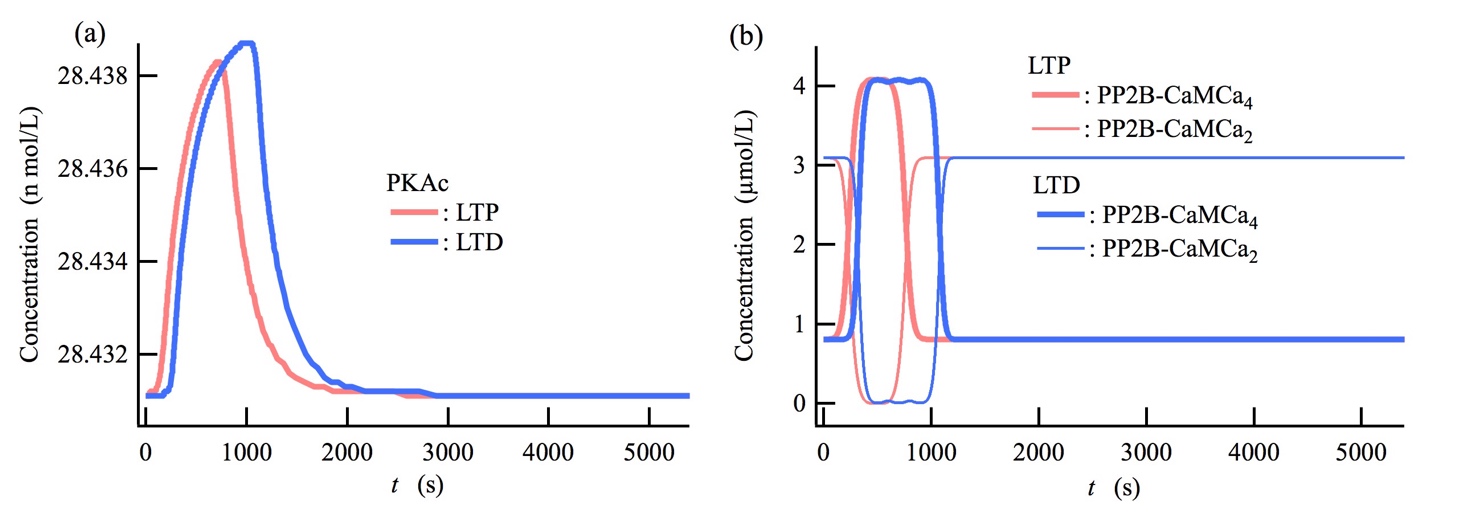
**

**Figure S7.** **The activation levels of PKA and PP2B are not effectively regulated by differences in Ca^2+^ concentration during LTP and LTD stimulation.** The competition between phosphorylation and dephosphorylation of GluA1 serine 845 is regulated by the activation level of PKA and PP2B. However, the LTP and LTD stimulation result in no obvious difference on the activation levels of PKA and PP2B, implying that the other calcium-dependent regulatory mechanism is needed to induce LTP and LTD expressions in addition to the previously proposed plasticity mechanism based only on the phosphorylation/dephosphorylation of GluA1 S845 [3].

**
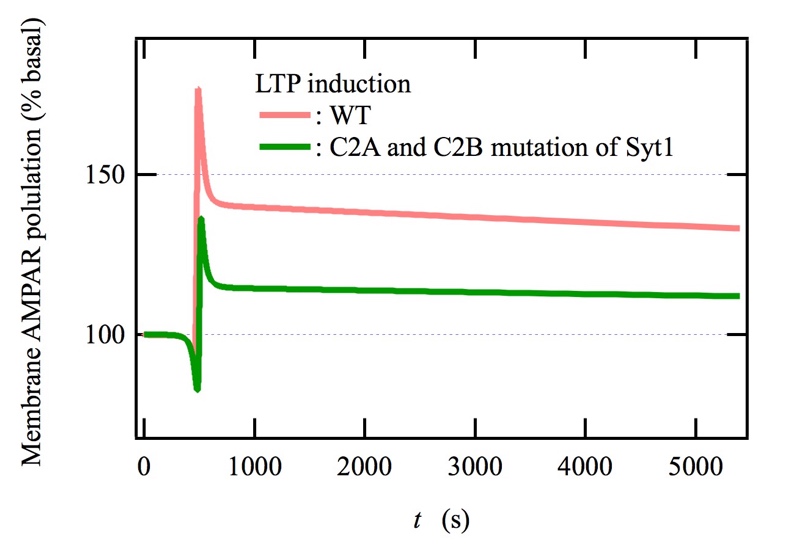
**

**Figure S8.** **Ca^2+^-binding site mutations of Syt1 in both the C2A and C2B domains block hippocampal LTP** [4]**.** Parameters for calcium-binding site mutations of Syt1 used here are provided in Supplementary Table S6.

**SUPPLEMENTARY INFORMATION METHOD**

**Model Construction**

**Ca^2+^ dynamics**

Ca^2+^ pulses are used to trigger hippocampal LTP and LTD induction. A model of Ca^2+^ dynamics is responsible for regulation of intracellular Ca^2+^ concentration ([Ca^2+^]) and comprises Ca^2+^ flux into the cytosol and extrusion of Ca^2+^ from the cytosol, thus plays an important role in the network model of hippocampal LTP and LTD inductions. The Ca^2+^ flux is used to elevate [Ca^2+^] and is modelled with a single Gaussian function for LTP stimulation and with a train of three Gaussian functions for LTD stimulation. The following equation

$f_{i}\left( t \right)=\frac{A_{i}}{\sqrt{2\pi\sigma_{i}^{2}}}\text{exp}\left[ \frac{-\left( t-t_{i} \right)^{2}}{2\sigma_{i}^{2}} \right]$

is employed as the *i* *th* Ca^2+^ pulse. We used the parameters shown in the table below for the LTP and LTD stimulation.

| LTP stimulation | Parameter values |
| --- | --- |
| $A_{1}$ | ﻿﻿275000 µmol/l |
| $\sigma_{1}$ | 100 s |
| $t_{1}$ | 500s |
| LTD stimulation |  |
| $A_{1}$ | ﻿140000 µmol/l |
| $\sigma_{1}$ | 70 s |
| $t_{1}$ | 500s |
| $A_{2}$ | ﻿140000 µmol/l |
| $\sigma_{2}$ | 70 s |
| $t_{2}$ | 700 s |
| $A_{3}$ | ﻿140000 µmol/l |
| $\sigma_{3}$ | 70 s |
| $t_{3}$ | 900 s |

To confirm how the Ca2+ influx modeling affects the result of LTP and LTD induction, we also examine a two-sigmoid function model to mimic the Ca^2+^ influx:

$f\left( t \right)=A_{sigmoid}\left\{ \frac{1}{1+exp\left[ -a\left( t-t_{s} \right) \right]}-\frac{1}{1+exp\left[ -a\left( t-t_{f} \right) \right]} \right\}$.

We used the parameters shown in the table below for LTP and LTD stimulation.

| LTP stimulation | Parameter values |
| --- | --- |
| $A_{sigmoid}$ | ﻿﻿1110 µmol/l |
| $a$ | 0.1s^-1^ |
| $t_{s}$ | 500s |
| $t_{f}$ | 600s |
| LTD stimulation |  |
| $A_{sigmoid}$ | ﻿820 µmol/l |
| $a$ | 0.1s^-1^ |
| $t_{s}$ | 500s |
| $t_{f}$ | 950s |

The model of Ca^2+^ extrusion used in this study (see below) is based on previous works [5-7]. We also introduced a constant flow of Ca^2+^ into the cytosol as a zero order reaction, which counteracts the extrusion of Ca^2+^ from the cytosol, so that the system sustain a basal Ca^2+^ concentration of ~ 50 nmol/L [6].

Ca + PMCA = CaPMCA

CaPMCA -> PMCA

Ca + NCX = CaNCX

CaNCX -> NCX

Ca + SERCA = CaSERCA

Ca + CaSERCA = Ca_2_SERCA

Ca_2_SERCA -> SERCA

Ca_leak_into: -> Ca (This work)

Ca_flux_into: -> Ca (This work)

**cAMP synthesis from ATP**

cAMP is synthesized by calcium-calmodulin bound adenylyl cyclase 1 (AC1). The related model used in this study (see below) is based on previous works [8,9].

CaM + 2 Ca = CaMCa_2_

CaMCa_2_ + 2 Ca = CaMCa_4_

AC1 + CaMCa_4_ = AC1-CaMCa_4_

AC1-CaMCa_4_ + ATP = AC1-CaMCa_4_-ATP

AC1-CaMCa_4_-ATP -> AC1-CaMCa_4_ + cAMP

**cAMP degradation into ATP**

cAMP is degraded through multiple pathways by phosphodiesterase type 1 (PDE1) [10,11], phosphodiesterase type 4 (PDE4) [12], and phosphorylated PDE4 (pPDE4) by PKA [13]. The models for these cAMP degradation reactions used in this study (shown below) are based on previous works [8,9].

PDE1 + CaMCa_4_ = PDE1-CaMCa_4_

PDE1-CaMCa_4_ + cAMP = PDE1-CaMCa_4_-cAMP

PDE1-CaMCa_4_-cAMP -> PDE1-CaMCa_4_ + AMP

AMP -> ATP

PDE4 + cAMP = PDE4-cAMP

PDE4-cAMP -> PDE4 + AMP

PDE4 + PKAc = PDE4-PKAc

PDE4-PKAc -> pPDE4 + PKAc

pPDE4 + cAMP = pPDE4-cAMP

pPDE4-cAMP -> pPDE4 + AMP

PDE4-cAMP + PKAc = PDE4-cAMP-PKAc

PDE4-cAMP-PKAc -> pPDE4-cAMP + PKAc

pPDE4 -> PDE4

**Phosphorylation of the S845 of GluA1 by cAMP-bound PKA**

PKA binds to A-kinase anchoring protein 150 (AKAP150) [14-16] and these form a signaling complex at the postsynaptic plasma membrane. PKA is activated by cAMP and the resulting PKA, namely, PKAc, phosphorylates the S845 of GluA1 at the postsynaptic membrane [17,18]. The model on the activation of PKA used in this study (shown below) is based on a previous work [8]. The model of the phosphorylation of GluA1 S845 used in this study (shown below) is also according to previous works [19,20].

PKA + 2 cAMP = PKAcAMP_2_

PKAcAMP_2_ + 2 cAMP = PKAcAMP_4_

PKAcAMP_4_ = R2_cAMP_4_ + 2 PKAc

R2_cAMP_4_ = R2 + 4 cAMP (The parameter obtained from [21])

R2 + 2 PKAc -> PKA (The parameters are adjusted in this work)

S845-GluR1 + PKAc = GluR1-PKAc

S845-GluR1-PKAc -> pS845-GluR1 + PKAc

**Dephosphorylation of the S845 of GluA1 by Ca^2+^-calmodulin-bound PP2B**

Protein phosphatase 2B (PP2B, also known as Calcineurin) binds to AKAP150 [14-16] and forms a signaling complex with AKAP150 at the postsynaptic plasma membrane. Calmodulin (CaM) has Ca^2+^-binding affinity and PP2B is activated by calcium-bound CaM [22]. The calcium-calmodulin-bound PP2B dephosphorylates the serine 845 of GluA1 at the postsynaptic plasma membrane [2]. The model of Ca^2+^-dependent activation of PP2B used in this study (shown below) is provided by previous works [20,22,23]. The model on the dephosphorylation of serine 845 GluA1 used in this study (shown below) is also presented by previous works [19,20].

CaM + 2 Ca = CaMCa_2_

CaMCa_2_ + 2 Ca = CaMCa_4_

PP2B + CaM = PP2B-CaM

PP2B + CaMCa_2_ = PP2B-CaMCa_2_

PP2B + CaMCa_4_ = PP2B-CaMCa_4_

PP2B-CaM + 2 Ca = PP2B-CaMCa_2_

PP2B-CaMCa_2_ + 2 Ca = PP2B-CaMCa_4_

pS845-GluR1 + PP2B-CaMCa_4_ = pS845-GluR1-PP2B-CaMCa_4_

pS845-GluR1-PP2B-CaMCa_4_ -> GluR1 + PP2B-CaMCa_4_

**Phosphorylation of the S880 of GluA2 by Ca^2+^-bound PKC**

Protein kinase C (PKC) also forms a signaling complex with the AKAP150 [14] at the postsynaptic plasma membrane. PKC has three C2 domains that bind Ca^2+^ ions [24], and Ca^2+^-bound PKC phosphorylates the S880 site of GluA2 at the postsynaptic membrane [25]. The model of Ca^2+^-binding to PKC used in this study (shown below) is provided by previous works [6,7,24]. The model of the phosphorylation of the S880 of GluA2 by Ca^2+^-bound PKC is according to a previous work [7].

PKC + Ca = CaPKC

CaPKC + Ca = Ca_2_PKC

Ca_2_PKC + Ca = Ca_3_PKC

S880-GluR2 + Ca_3_PKC = S880-GluR2-Ca_3_PKC

S880-GluR2-Ca_3_PKC -> pS880-GluR2 + Ca_3_PKC

**PICK1 that triggers endocytosis of AMPARs**

Protein interacting with C-kinase 1 (PICK1) is a Ca^2+^-sensor protein that has two Ca^2+^-binding sites and Ca^2+^-bound PICK1 triggers endocytosis of AMPARs by binding to phosphorylated S880 of GluA2 [25-27]. The Ca^2+^-binding constants, *K*_1b_ and *K*_2b_, have been experimentally determined to be 1.80 and 0.27 L/µmol, respectively [28]. The rate constants for the forward and backward reaction shown below can be modeled using *K*_1b_ and *K*_2b_.

PICK1 + Ca = CaPICK1 *k*_1f_, *k*_1b_

CaPICK1 + Ca = Ca_2_PICK1 *k*_2f_, *k*_2b_

If we assumed the forward reaction constants, *k*_1f_ and *k*_2f_, to be 21.6 and 10.8 L/(µmol s), respectively, the backward reaction constants, *k*_1b_ and *k*_2b_, were determined to be 12 and 40 s^-1^, respectively, by using *k*_1b_ = *k*_1f_/*K*_1b_ and *k*_2b_ = *k*_2f_/*K*_2b_.

**Syt1 that triggers exocytosis of AMPARs**

Synaptic vesicle protein, synaptotagmin 1 (Syt1) is a Ca^2+^-sensor protein that has two Ca^2+^-binding domains, C2A and C2B [29]. The Ca^2+^-bound Syt1 triggers exocytosis of AMPARs by cooperating with synaptic vesicle protein synaptobrevin-2/VAMP2, synaptic membrane protein synaptotagmin 7 (Syt7), and amongst others [4,30-33]. In fact, it has been observed that Ca^2+^-binding site mutations of Syt1 in both the C2A and C2B domains block hippocampal LTP [4]. Therefore, it would be appropriate to use Ca^2+^-binding constants of Syt1 to model a Ca^2+^-dependent regulating factor on the exocytosis mediated by Syt1 together with Syt7, synaptobrevin-2/VAMP2, and complexin, amongst others [4,29,31-34]. The C2A and C2B domain of Syt1 bind three and two Ca^2+^ ions, respectively. Ca^2+^-binding constants for the C2A domain, *K*_A1_, *K*_A2_, and *K*_A3_, have been experimentally determined to be 2.04 x 10^-2^, 2.04 x 10^-3^, and 3.21 x 10^-4^ L/µmol, respectively, and that for the C2B domain, *K*_B1_, has been determined to be 7.04 x 10^-3^ L/µmol [29]. The reaction equations and each forward and backward rate constant are defined as follows:

Syt1 + Ca = (Ca)_A_Syt1 *k*_A1f_, *k*_A1b_

(Ca)_A_Syt1 + Ca = (Ca_2_)_A_Syt1 *k*_A2f_, *k*_A2b_

(Ca_2_)_A_Syt1 + Ca = (Ca_2_)_A_(Ca)_B_Syt1 *k*_B3f_, *k*_B3b_

(Ca_2_)_A_(Ca)_B_Syt1 + Ca = (Ca_2_)_A_(Ca_2_)_B_Syt1 *k*_B4f_, *k*_B4b_

(Ca_2_)_A_(Ca_2_)_B_Syt1 + Ca = (Ca_3_)_A_(Ca_2_)_B_Syt1 *k*_A5f_, *k*_A5b_

If we assumed these forward rate constants, *k*_A1f_, *k*_A2f_, *k*_B3f_, *k*_B4f_, and *k*_A5f_, to be 2.0, 1.0, 1.0, 1.0, and 0.5 L/(µmol s), respectively, those backward rate constants, *k*_A1b_, *k*_A2b_, *k*_B3b_, *k*_B4b_, and *k*_A5b_, were determined to be 97, 488, 142, 142, and 1560 s^-1^, respectively, by using the experimentally determined binding constants, *K*_A1_, *K*_A2_, *K*_A3_, and *K*_B1_.

**Network model on phosphorylation/dephosphorylation dynamics of AMPARs at the synaptic membrane**

We incorporated GluA1/A2 heterotetramer (Fig. 1a), which is the most dominant subtype at hippocampal neurons [35], as the AMPAR model. The state of the AMPAR is identified by the 2 x 2 matrix shown in Fig. S9. The upper left and right element indicate the numbers of phosphorylated S845 of GluA1 and of phosphorylated S880 of GluA2, respectively. The lower left and right element indicate the number of synaptic associated protein 97 kDa (SAP97) [15] bound to phosphorylated GluA1 and the number of PICK1 bound to phosphorylated GluA2, respectively. If the number of lower right element is 0, 1, and 2, the number of glutamate receptor interacting protein 1 (GRIP1) [15,36] bound to dephosphorylated GluA2 corresponds to 2, 1, and 0, respectively. State transitions occur between states of neighbour phosphorylation levels under keeping the number of AMPAR interaction proteins and between neighbour states inside the same phosphorylation level. The state of AMPARs that are incorporated into the postsynaptic membrane by Sty1-mediated exocytosis corresponds to the state with the phosphorylated levels of both GluA1 and GluA2 being zero and without SAP97 but with GRIP1 (the bottom left panel in Fig. S9). The endocytosis of AMPARs that is mediated by PICK1 occurs through three states at the phosphorylated level of the bottom right (Fig. S9).


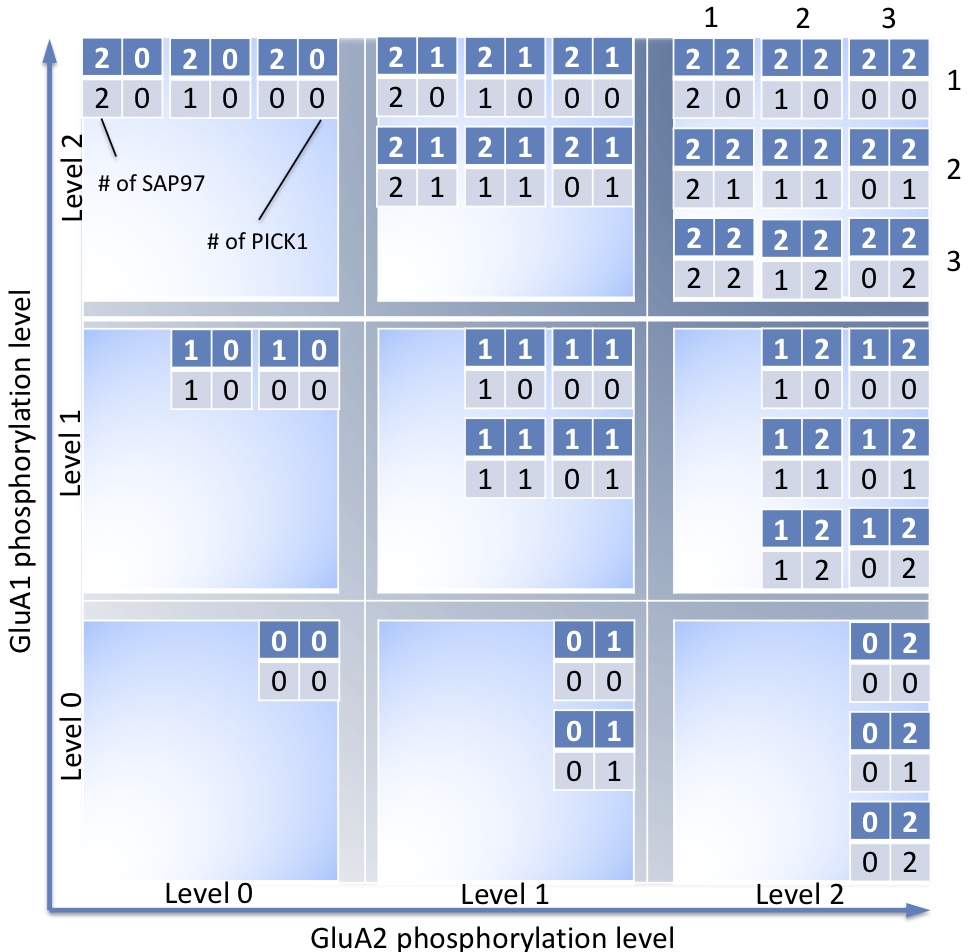


**Figure S9.** **Network model on phosphorylation/dephosphorylation dynamics of AMPARs at the synaptic membrane**.

**Network model on the dynamics of PICK1-binding to AMPARs and of Ca^2+^-binding to PICK1-bond AMPARs**

In addition to PICK1 binding to AMPARs, which is already considered in Fig. S9, we also take into consideration CaPICK1-binding and Ca_2_PICK1-binding to the AMPARs with two dephosphorylated GluA1s and two phosphorylated GluA2s (i.e., the AMPARs with three states at the phosphorylated level of the bottom right in Fig. S10), and also take into consideration Ca^2+^-binding to the PICK1-bound AMPARs (Fig. S10). Three examples showing the correspondences between the 2 x 2 and 1 x 3 matrix representations are provided at the top of Fig. S10. All the possible state transitions for Ca^2+^-binding and PICK1-binding due to the states listed at the left-hand side of Fig. S10 are given as horizontal transitions in Fig. S10. To satisfy the detailed balance condition, we assume all the rates of PICK1-, CaPICK1-, and Ca_2_PICK1-binding to AMPAR to be equivalent and also assume all the rates of PICK1-, CaPICK1-, and Ca_2_PICK1-dissociation from AMPAR to be equivalent.


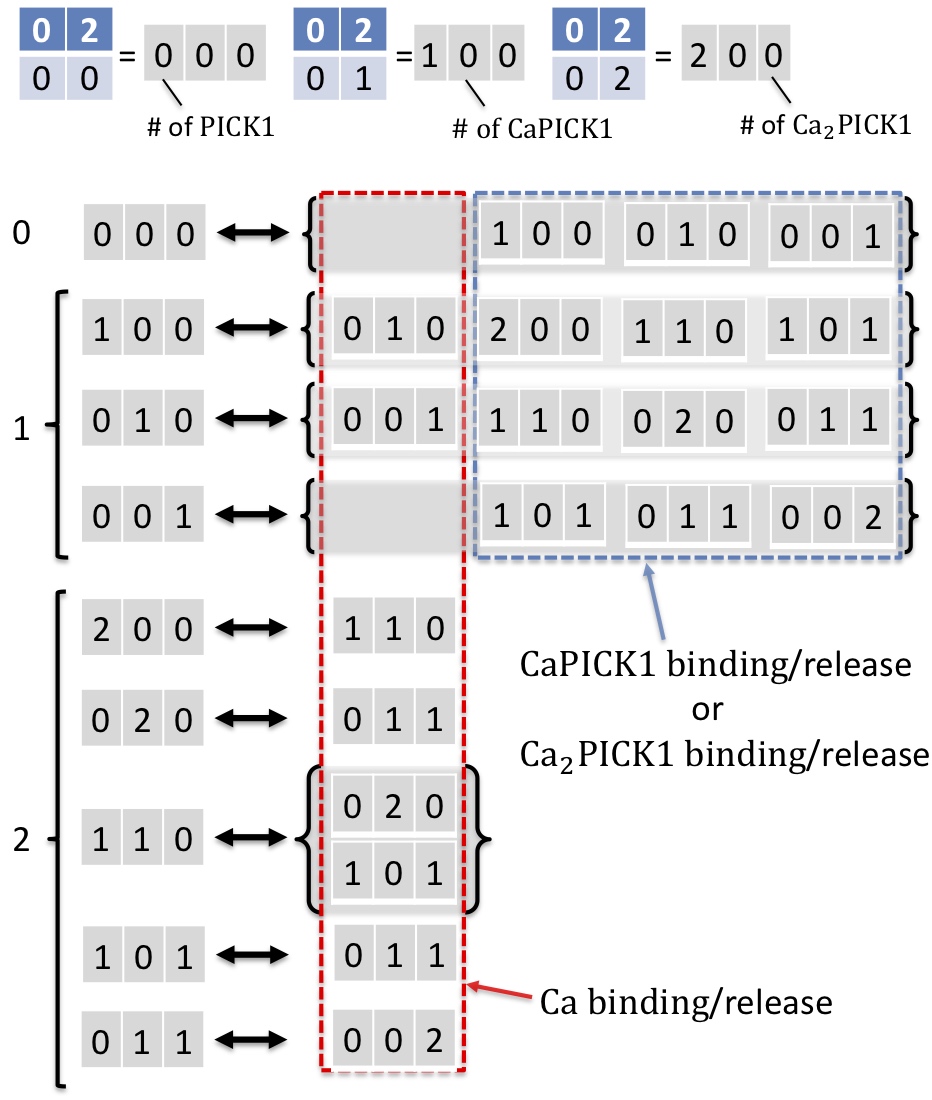


**Figure S10.** **Network model on the dynamics of PICK1-binding to AMPARs and of Ca^2+^-binding to PICK1-bond AMPARs.** Here, the AMPARs with two dephosphorylated GluA1 and two phosphorylated GluA2, namely, the AMPARs with three states at the phosphorylated level of the bottom right in Fig. S9, are taken into consideration. Three examples showing the correspondences between the 2 x 2 and 1 x 3 matrix representations are given at the top of Fig. S10.

**Network model on dephosphorylation dynamics of GluA2 that regulates PICK1 dissociation from endocytic AMPARs and GRIP1-binding to those in the cytosol**

Three kinds of one PICK1-bound AMPAR and six kinds of two PICK1-bound AMPAR, which appear at Fig. S10, are internalized as recycling endosomes by PICK1-mediated endocytosis (these are indicated with the blue and red box on the bottom left of Fig. S11). We assume that the serine 880 of GluA2 of internalized AMPARs is dephosphorylated on the recycling endosomes by protein phosphatase 2A (PP2A) [7]. PICK1s that bind to the GluA2 are dissociated and instead GRIP1 binds to the dephosphorylated S880 of GluA2 [15,36]. In the present study, we assume that the recycling endosomes containing the AMPARs with the state at the upper right of Fig. S11 are transported by molecular motor myosin V_b_ toward the postsynaptic membrane [37-39].


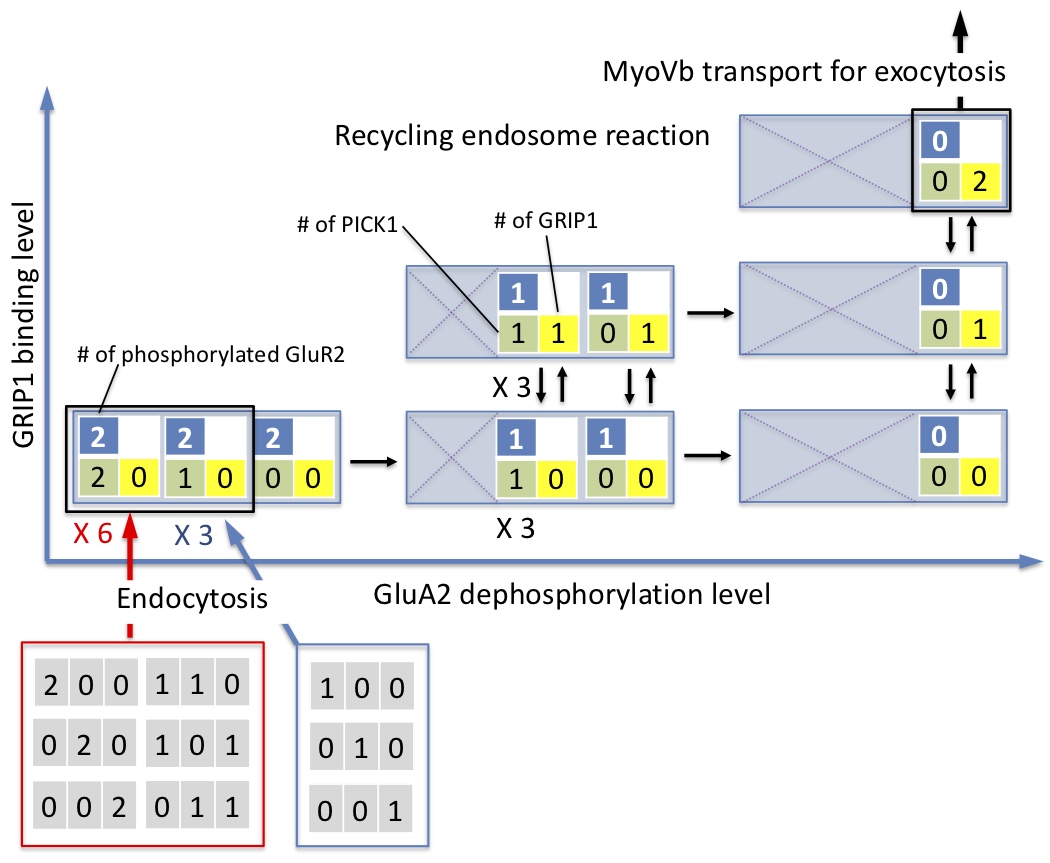


**Figure S11.** **Network model on dephosphorylation dynamics of GluA2 that regulates PICK1-dissociation from endocytic AMPARs and GRIP1-binding to those in the cytosol.**

**An approximate treatment on the relation between recycling endosome and Syt1**

Ca^2+^-sensor synaptic vesicle protein synaptotagmin 1 (Syt1) mediates exocytosis of AMPARs, and the AMPARs are internalized into the cytosol as recycling endosomes. The recycling endosomes containing AMPARs undergo diffusion in the cytosol and those bound to molecular motor myosin V_b_ are transported to the peri-synaptic and synaptic membrane surface. On the basis of an analogy with Syt1-mediated synaptic vesicle exocytosis at peri-synaptic and synaptic membrane that results in neurotransmitter release [4,30-33], the Syt1 proteins are expected to be carried by the endocytic vesicles along with AMPARs in postsynaptic neurons as well. However, the network model becomes extremely complicated in the cytosol, if multiple Ca^2+^-binding Syt1 species are included in the recycling endosomes together with AMPARs with various states. In the present work, for simplification, we introduce the following approximation: the Syt1 proteins are artificially remained at the postsynaptic membrane when AMPARs are internalized as the recycling endosomes; the Syt1 proteins left at the postsynaptic membrane bind to the recycling endosomes as immediately as myosin-V_b_ molecules transport the recycling endosomes to peri-synaptic and synaptic membrane surface.

**SUPPLEMENTARY INFORMATION TABLE**

**Table S1. Parameters of the network model at the initial condition.** For simplification of making input data, we set the initial concentrations for the other species that are not provided in this table and all the simulations are started from the steady state condition.

|  | **Volume (l)** | **Ref.** |
| --- | --- | --- |
| Compartment size | 6.00E-17 | [7,40] |
| **Species** | **Initial concentration (µmol/l)** |  |
| AC1 | 1.6686 | [8] |
| AC1-CaMCa_4_ | 0.14 | This work |
| AC1-CaMCa_4_-ATP | 0.1422 | [8] |
| AMP | 0.495 | [8] |
| ATP | 1998.64 | [8] |
| Ca | 0.05 | [7] |
| CaM | 8.775 | [8] |
| CaMCa_2_ | 0.291 | [8] |
| CaMCa_4_ | 0.01 | [8] |
| cAMP | 0.027 | [8] |
| MyoV | 10 | This work |
| NCX | 0.111 | [7] |
| PDE1 | 3.457 | [8] |
| PDE1-CaMCa_4_ | 0.489 | [8] |
| PDE1-CaMCa_4_-cAMP | 0.001 | [8] |
| PDE4 | 2.766 | [8] |
| PDE4-cAMP | 0.034 | [8] |
| PDE4-PKAc | 0.025 | [8] |
| PICK1 | 1 | This work |
| PKA | 1.7634 | [8] |
| PKAcAMP_2_ | 0.3426 | [8] |
| PKAcAMP_4_ | 0.0102 | [8] |
| PKC | 3 | This work |
| PMCA | 0.277 | [7] |
| PP2A | 1.1 | This work |
| PP2B-CaM | 2.99 | [8] |
| PP2B-CaMCa_2_ | 0.989 | [8] |
| PP2B-CaMCa_4_ | 0.1 | [8] |
| pPDE4 | 0.122 | [8] |
| pPDE4-cAMP | 0.001 | [8] |
| R1R2(GRIP_2_) | 2 | This work |
| R2_cAMP_4_ | 0.0096 | This work |
| SAP | 4 | This work |
| SERCA | 1.66 | [7] |
| Syt1 | 4 | This work |

**Table S2. Reactions and parameters of the network model on hippocampal bidirectional synaptic plasticity.**

| **No.** | **Reaction** | ***k*_f_** | ***k*_b_** | **Ref.** |
| --- | --- | --- | --- | --- |
| 1 | Syt1 + Ca = (Ca)_A_Syt1 | 2 l/(µmol*s) | 97.6 1/s | This work |
| 2 | (Ca_2_)_A_Syt1 + Ca = (Ca_2_)_A_(Ca)_B_Syt1 | 1 l/(µmol*s) | 142 1/s | This work |
| 3 | (Ca_2_)_A_(Ca)_B_Syt1 + Ca = (Ca_2_)_A_(Ca_2_)_B_Syt1 | 1 l/(µmol*s) | 142 1/s | This work |
| 4 | (Ca)_A_Syt1 + Ca = (Ca_2_)_A_Syt1 | 1 l/(µmol*s) | 488 1/s | This work |
| 5 | (Ca_2_)_A_(Ca_2_)_B_Syt1 + Ca = (Ca_3_)_A_(Ca_2_)_B_Syt1 | 0.5 l/(µmol*s) | 1560 1/s | This work |
| 6 | AC1 + CaMCa_4_ = AC1-CaMCa_4_ | 6 l/(µmol*s) | 0.9 1/s | [8,9,41] |
| 7 | PDE4-cAMP -> PDE4 + AMP | 17.233 1/s |  | [8,9,12] |
| 8 | AMP -> ATP | 1.0 1/s |  | [8] |
| 9 | PDE1-CaMCa_4_-cAMP -> PDE1-CaMCa_4_ + AMP | 11 1/s |  | [8,42] |
| 10 | pPDE4-cAMP -> pPDE4 + AMP | 34.467 1/s |  | [8,13] |
| 11 | -> Ca | 14 µmol/(l*s) |  | This work |
| 12 | R1R2(pS_2_-PICK1) + Ca = R1R2(pS_2_-CaPICK1) | 21.6 l/(µmol*s) | 12 1/s | Detailed balance condition (DBC) based on Eq. 31 |
| 13 | R1R2(pS_2_-PICK1-PICK1) + Ca = R1R2(pS_2_-PICK1-CaPICK1) | 21.6 l/(µmol*s) | 12 1/s | DBC based on Eq. 31 |
| 14 | R1R2(pS_2_-PICK1-CaPICK1) + Ca = R1R2(pS_2_-CaPICK1-CaPICK1) | 21.6 l/(µmol*s) | 12 1/s | DBC based on Eq. 31 |
| 15 | R1R2(pS_2_-PICK1-Ca_2_PICK1) + Ca = R1R2(pS_2_-CaPICK1-Ca_2_PICK1) | 21.6 l/(µmol*s) | 12 1/s | DBC based on Eq. 31 |
| 16 | R1R2(pS_2_-CaPICK1) + Ca = R1R2(pS_2_-Ca_2_PICK1) | 10.8 l/(µmol*s) | 40 1/s | DBC based on Eq. 20 |
| 17 | R1R2(pS_2_-CaPICK1-CaPICK1) + Ca = R1R2(pS_2_-CaPICK1-Ca_2_PICK1) | 10.8 l/(µmol*s) | 40 1/s | DBC based on Eq. 20 |
| 18 | R1R2(pS_2_-PICK1-CaPICK1) + Ca = R1R2(pS_2_-PICK1-Ca_2_PICK1) | 10.8 l/(µmol*s) | 40 1/s | DBC based on Eq. 20 |
| 19 | R1R2(pS_2_-CaPICK1-Ca_2_PICK1) + Ca = R1R2(pS_2_-Ca_2_PICK1-Ca_2_PICK1) | 10.8 l/(µmol*s) | 40 1/s | DBC based on Eq. 20 |
| 20 | CaPICK1 + Ca = Ca_2_PICK1 | 10.8 l/(µmol*s) | 40 1/s | This work |
| 21 | R1R2(pS_2_) + Ca_2_PICK1 = R1R2(pS_2_-Ca_2_PICK1) | 2.8 l/(µmol*s) | 5 1/s | [43] |
| 22 | R1R2(pS_2_-PICK1) + Ca_2_PICK1 = R1R2(pS_2_-PICK1-Ca_2_PICK1) | 2.8 l/(µmol*s) | 5 1/s | DBC based on Eq. 21 |
| 23 | R1R2(pS_2_-CaPICK1) + Ca_2_PICK1 = R1R2(pS_2_-CaPICK1-Ca_2_PICK1) | 2.8 l/(µmol*s) | 5 1/s | DBC based on Eq. 21 |
| 24 | R1R2(pS_2_-Ca_2_PICK1) + Ca_2_PICK1 = R1R2(pS_2_-Ca_2_PICK1-Ca_2_PICK1) | 2.8 l/(µmol*s) | 5 1/s | DBC based on Eq. 21 |
| 25 | CaPKC + Ca = Ca_2_PKC | 45.45 l/(µmol*s) | 40 1/s | [6,7,24,44] |
| 26 | Ca_2_PKC + Ca = Ca_3_PKC | 45.45 l/(µmol*s) | 40 1/s | [6,7,24,44] |
| 27 | 2 * Ca + CaM = CaMCa_2_ | 6000 l²/(µmol²*s) | 9.1 1/s | [8,45] |
| 28 | 2 * Ca + CaMCa_2_ = CaMCa_4_ | 100000 l²/(µmol²*s) | 1000 1/s | [8,46] |
| 29 | AC1-CaMCa_4_ + ATP = AC1-CaMCa_4_-ATP | 10 l/(µmol*s) | 2273 1/s | [8,47] |
| 30 | AC1-CaMCa4-ATP -> AC1-CaMCa4 + cAMP | 5.684 1/s |  | [8,47] |
| 31 | PICK1 + Ca = CaPICK1 | 21.6 l/(µmol*s) | 12 1/s | This work |
| 32 | R1R2(pS_2_) + CaPICK1 = R1R2(pS_2_-CaPICK1) | 2.8 l/(µmol*s) | 5 1/s | [26,48,49] |
| 33 | R1R2(pS_2_-PICK1) + CaPICK1 = R1R2(pS_2_-PICK1-CaPICK1) | 2.8 l/(µmol*s) | 5 1/s | DBC based on Eq. 32 |
| 34 | R1R2(pS_2_-CaPICK1) + CaPICK1 = R1R2(pS_2_-CaPICK1-CaPICK1) | 2.8 l/(µmol*s) | 5 1/s | DBC based on Eq. 32 |
| 35 | R1R2(pS_2_-Ca_2_PICK1) + CaPICK1 = R1R2(pS_2_-CaPICK1-Ca_2_PICK1) | 2.8 l/(µmol*s) | 5 1/s | DBC based on Eq. 32 |
| 36 | PKC + Ca = CaPKC | 1111 l/(µmol*s) | 12 1/s | [6,7,24,44] |
| 37 | R1R2endo(pS_2_-Ca_2_PICK1-Ca_2_PICK1) = R1R2endo(pS_2_-Ca_2_PICK1) + Ca_2_PICK1 | 5 1/s | 0.7 l/(µmol*s) | [48] |
| 38 | R1R2endo(pS_2_-CaPICK1-Ca_2_PICK1) = R1R2endo(pS_2_-CaPICK1) + Ca_2_PICK1 | 5 1/s | 0.7 l/(µmol*s) | DBC based on Eq. 37 |
| 39 | R1R2endo(pS_2_-PICK1-Ca_2_PICK1) = R1R2endo(pS_2_-PICK1) + Ca_2_PICK1 | 5 1/s | 0.7 l/(µmol*s) | DBC based on Eq. 37 |
| 40 | R1R2endo(pS_2_-Ca_2_PICK1) = R1R2endo(pS_2_) + Ca_2_PICK1 | 5 1/s | 0.7 l/(µmol*s) | DBC based on Eq. 37 |
| 41 | R1R2endo(pS-Ca_2_PICK1) = R1R2endo(pS) + Ca_2_PICK1 | 5 1/s | 0.7 l/(µmol*s) | DBC based on Eq. 37 |
| 42 | R1R2endo(GRIP-pS-Ca_2_PICK1) = R1R2endo(GRIP-pS) + Ca_2_PICK1 | 5 1/s | 0.7 l/(µmol*s) | DBC based on Eq. 37 |
| 43 | R1R2endo(pS_2_-CaPICK1-Ca_2_PICK1) = R1R2endo(pS_2_-Ca_2_PICK1) + CaPICK1 | 5 1/s | 0.7 l/(µmol*s) | DBC based on Eq. 37 |
| 44 | R1R2endo(pS_2_-CaPICK1-CaPICK1) = R1R2endo(pS_2_-CaPICK1) + CaPICK1 | 5 1/s | 0.7 l/(µmol*s) | DBC based on Eq. 37 |
| 45 | R1R2endo(pS_2_-PICK1-CaPICK1) = R1R2endo(pS_2_-PICK1) + CaPICK1 | 5 1/s | 0.7 l/(µmol*s) | DBC based on Eq. 37 |
| 46 | R1R2endo(pS_2_-CaPICK1) = R1R2endo(pS_2_) + CaPICK1 | 5 1/s | 0.7 l/(µmol*s) | DBC based on Eq. 37 |
| 47 | R1R2endo(pS-CaPICK1) = R1R2endo(pS) + CaPICK1 | 5 1/s | 0.7 l/(µmol*s) | DBC based on Eq. 37 |
| 48 | R1R2endo(GRIP-pS-CaPICK1) = R1R2endo(GRIP-pS) + CaPICK1 | 5 1/s | 0.7 l/(µmol*s) | DBC based on Eq. 37 |
| 49 | R1R2endo(pS-Ca_2_PICK1) + GRIP = R1R2endo(GRIP-pS-Ca_2_PICK1) | 5.5 l/(µmol*s) | 0.3 1/s | [48,50,51] |
| 50 | R1R2endo(pS-CaPICK1) + GRIP = R1R2endo(GRIP-pS-CaPICK1) | 5.5 l/(µmol*s) | 0.3 1/s | DBC based on Eq. 49 |
| 51 | R1R2endo(pS-PICK1) + GRIP = R1R2endo(GRIP-pS-PICK1) | 5.5 l/(µmol*s) | 0.3 1/s | DBC based on Eq. 49 |
| 52 | R1R2endo(pS) + GRIP = R1R2endo(GRIP-pS) | 5.5 l/(µmol*s) | 0.3 1/s | DBC based on Eq. 49 |
| 53 | R1R2endo + GRIP = R1R2endo(GRIP) | 5.5 l/(µmol*s) | 0.3 1/s | DBC based on Eq. 49 |
| 54 | R1R2endo(GRIP) + GRIP = R1R2endo(GRIP_2_) | 5.5 l/(µmol*s) | 0.3 1/s | DBC based on Eq. 49 |
| 55 | R1R2endo(pS_2_-PICK1-Ca_2_PICK1) = R1R2endo(pS_2_-Ca_2_PICK1) + PICK1 | 5 1/s | 0.7 l/(µmol*s) | [48] |
| 56 | R1R2endo(pS_2_-PICK1-CaPICK1) = R1R2endo(pS_2_-CaPICK1) + PICK1 | 5 1/s | 0.7 l/(µmol*s) | DBC based on Eq. 55 |
| 57 | R1R2endo(pS_2_-PICK1-PICK1) = R1R2endo(pS_2_-PICK1) + PICK1 | 5 1/s | 0.7 l/(µmol*s) | DBC based on Eq. 55 |
| 58 | R1R2endo(pS_2_-PICK1) = R1R2endo(pS_2_) + PICK1 | 5 1/s | 0.7 l/(µmol*s) | DBC based on Eq. 55 |
| 59 | R1R2endo(pS-PICK1) = R1R2endo(pS) + PICK1 | 5 1/s | 0.7 l/(µmol*s) | DBC based on Eq. 55 |
| 60 | R1R2endo(GRIP-pS-PICK1) = R1R2endo(GRIP-pS) + PICK1 | 5 1/s | 0.7 l/(µmol*s) | DBC based on Eq. 55 |
| 61 | R1R2endo(pS_2_-PICK1) + PP2A = R1R2endo(pS_2_-PICK1)PP2A | 0.08 l/(µmol*s) | 0.8 1/s | [7] |
| 62 | R1R2endo(GRIP-pS-CaPICK1) + PP2A = R1R2endo(GRIP-pS-CaPICK1)PP2A | 0.08 l/(µmol*s) | 0.8 1/s | DBC based on Eq. 61 |
| 63 | R1R2endo(GRIP-pS-PICK1) + PP2A = R1R2endo(GRIP-pS-PICK1)PP2A | 0.08 l/(µmol*s) | 0.8 1/s | DBC based on Eq. 61 |
| 64 | R1R2endo(GRIP-pS) + PP2A = R1R2endo(GRIP-pS)PP2A | 0.08 l/(µmol*s) | 0.8 1/s | DBC based on Eq. 61 |
| 65 | R1R2endo(pS_2_-CaPICK1) + PP2A = R1R2endo(pS_2_-CaPICK1)PP2A | 0.08 l/(µmol*s) | 0.8 1/s | DBC based on Eq. 61 |
| 66 | R1R2endo(pS_2_-Ca2PICK1) + PP2A = R1R2endo(pS_2_-Ca_2_PICK1)PP2A | 0.08 l/(µmol*s) | 0.8 1/s | DBC based on Eq. 61 |
| 67 | R1R2endo(pS_2_) + PP2A = R1R2endo(pS_2_)PP2A | 0.08 l/(µmol*s) | 0.8 1/s | DBC based on Eq. 61 |
| 68 | R1R2endo(pS-Ca_2_PICK1) + PP2A = R1R2endo(pS-Ca_2_PICK1)PP2A | 0.08 l/(µmol*s) | 0.8 1/s | DBC based on Eq. 61 |
| 69 | R1R2endo(pS-CaPICK1) + PP2A = R1R2endo(pS-CaPICK1)PP2A | 0.08 l/(µmol*s) | 0.8 1/s | DBC based on Eq. 61 |
| 70 | R1R2endo(pS-CaPICK1) + PP2A = R1R2endo(pS-CaPICK1)PP2A | 0.08 l/(µmol*s) | 0.8 1/s | DBC based on Eq. 61 |
| 71 | R1R2endo(pS) + PP2A = R1R2endo(pS)PP2A | 0.08 l/(µmol*s) | 0.8 1/s | DBC based on Eq. 61 |
| 72 | R1R2endo(GRIP-pS-Ca_2_PICK1) + PP2A = R1R2endo(GRIP-pS-Ca_2_PICK1)PP2A | 0.08 l/(µmol*s) | 0.8 1/s | DBC based on Eq. 61 |
| 73 | R1R2endo(pS_2_-PICK1)PP2A -> R1R2endo(pS-PICK1) + PP2A | 2 1/s |  | [7] |
| 74 | R1R2endo(GRIP-pS-CaPICK1)PP2A -> R1R2endo(GRIP) + CaPICK1 + PP2A | 2 1/s |  | [7] |
| 75 | R1R2endo(GRIP-pS-PICK1)PP2A -> R1R2endo(GRIP) + PICK1 + PP2A | 2 1/s |  | [7] |
| 76 | R1R2endo(GRIP-pS)PP2A -> R1R2endo(GRIP) + PP2A | 2 1/s |  | [7] |
| 77 | R1R2endo(pS_2_-CaPICK1)PP2A -> R1R2endo(pS-CaPICK1) + PP2A | 2 1/s |  | [7] |
| 78 | R1R2endo(pS_2_-Ca_2_PICK1)PP2A -> R1R2endo(pS-Ca_2_PICK1) + PP2A | 2 1/s |  | [7] |
| 79 | R1R2endo(pS_2_)PP2A -> R1R2endo(pS) + PP2A | 2 1/s |  | [7] |
| 80 | R1R2endo(pS-Ca_2_PICK1)PP2A -> R1R2endo + Ca_2_PICK1 + PP2A | 2 1/s |  | [7] |
| 81 | R1R2endo(pS-CaPICK1)PP2A -> R1R2endo + CaPICK1 + PP2A | 2 1/s |  | [7] |
| 82 | R1R2endo(pS-PICK1)PP2A -> R1R2endo + PICK1 + PP2A | 2 1/s |  | [7] |
| 83 | R1R2endo(pS)PP2A -> R1R2endo + PP2A | 2 1/s |  | [7] |
| 84 | R1R2endo(GRIP-pS-Ca_2_PICK1)PP2A -> R1R2endo(GRIP) + Ca_2_PICK1 + PP2A | 2 1/s |  | [7] |
| 85 | R1R2(pS_2_-Ca_2_PICK1) -> R1R2endo(pS_2_-Ca_2_PICK1) | 0.001 1/s |  | This work |
| 86 | R1R2(pS_2_-Ca_2_PICK1-Ca_2_PICK1) -> R1R2endo(pS_2_-Ca_2_PICK1-Ca_2_PICK1) | 0.06 1/s |  | This work |
| 87 | R1R2(pS_2_-CaPICK1) -> R1R2endo(pS_2_-CaPICK1) | 0.0005 1/s |  | This work |
| 88 | R1R2(pS_2_-CaPICK1-Ca_2_PICK1) -> R1R2endo(pS_2_-CaPICK1-Ca_2_PICK1) | 0.02 1/s |  | This work |
| 89 | R1R2(pS_2_-CaPICK1-CaPICK1) -> R1R2endo(pS_2_-CaPICK1-CaPICK1) | 0.001 1/s |  | This work |
| 90 | R1R2(pS_2_-PICK1) -> R1R2endo(pS_2_-PICK1) | 8e-05 1/s |  | This work |
| 91 | R1R2(pS_2_-PICK1-Ca_2_PICK1) -> R1R2endo(pS_2_-PICK1-Ca_2_PICK1) | 0.001 1/s |  | This work |
| 92 | R1R2(pS_2_-PICK1-CaPICK1) -> R1R2endo(pS_2_-PICK1-CaPICK1) | 0.0002 1/s |  | This work |
| 93 | R1R2(pS_2_-PICK1-PICK1) -> R1R2endo(pS_2_-PICK1-PICK1) | 0.00016 1/s |  | This work |
| 94 | Ca + NCX = Ca_NCX | 800 l/(µmol*s) | 100 1/s | [6,7] |
| 95 | Ca_NCX -> NCX | 2300 1/s |  | [6,7] |
| 96 | PDE1 + CaMCa_4_ = PDE1-CaMCa_4_ | 100 l/(µmol*s) | 1 1/s | [8-10] |
| 97 | PDE1-CaMCa_4_ + cAMP = PDE1-CaMCa_4_-cAMP | 4.6 l/(µmol*s) | 44 1/s | [8,9,42] |
| 98 | PDE4 + cAMP = PDE4-cAMP | 21.66 l/(µmol*s) | 68.95 1/s | [8,9,12] |
| 99 | PDE4-cAMP + PKAc = PDE4-cAMP-PKAc | 0.428 l/(µmol*s) | 0.56 1/s | [8,9,13] |
| 100 | PDE4 + PKAc = PDE4-PKAc | 0.428 l/(µmol*s) | 0.56 1/s | [8,9,13] |
| 101 | R1R2(GRIP-pS) + PICK1 = R1R2(GRIP-pS-PICK1) | 2.8 l/(µmol*s) | 5 1/s | [48] |
| 102 | R1(pS-SAP)R2(pS_2_) + PICK1 = R1(pS-SAP)R2(pS_2_-PICK1) | 2.8 l/(µmol*s) | 5 1/s | DBC based on Eq. 101 |
| 103 | R1(pS)R2(pS_2_-PICK1) + PICK1 = R1(pS)R2(pS_2_-PICK1-PICK1) | 2.8 l/(µmol*s) | 5 1/s | DBC based on Eq. 101 |
| 104 | R1(pS-SAP)R2(pS_2_-PICK1) + PICK1 = R1(pS-SAP)R2(pS_2_-PICK1-PICK1) | 2.8 l/(µmol*s) | 5 1/s | DBC based on Eq. 101 |
| 105 | R1(pS_2_)R2(pS_2_) + PICK1 = R1(pS_2_)R2(pS_2_-PICK1) | 2.8 l/(µmol*s) | 5 1/s | DBC based on Eq. 101 |
| 106 | R1(pS_2_-SAP)R2(pS_2_) + PICK1 = R1(pS_2_-SAP)R2(pS_2_-PICK1) | 2.8 l/(µmol*s) | 5 1/s | DBC based on Eq. 101 |
| 107 | R1(pS_2_-SAP_2_)R2(pS_2_) + PICK1 = R1(pS_2_-SAP_2_)R2(pS_2_-PICK1) | 2.8 l/(µmol*s) | 5 1/s | DBC based on Eq. 101 |
| 108 | R1(pS_2_)R2(pS_2_-PICK1) + PICK1 = R1(pS_2_)R2(pS_2_-PICK1-PICK1) | 2.8 l/(µmol*s) | 5 1/s | DBC based on Eq. 101 |
| 109 | R1(pS_2_-SAP)R2(pS_2_-PICK1) + PICK1 = R1(pS_2_-SAP)R2(pS_2_-PICK1-PICK1) | 2.8 l/(µmol*s) | 5 1/s | DBC based on Eq. 101 |
| 110 | R1(pS_2_-SAP_2_)R2(pS_2_-PICK1) + PICK1 = R1(pS_2_-SAP_2_)R2(pS_2_-PICK1-PICK1) | 2.8 l/(µmol*s) | 5 1/s | DBC based on Eq. 101 |
| 111 | R1(pS_2_-SAP_2_)R2(pS_2_-PICK1) + PICK1 = R1(pS_2_-SAP_2_)R2(pS_2_-PICK1-PICK1) | 2.8 l/(µmol*s) | 5 1/s | DBC based on Eq. 101 |
| 112 | R1(pS)R2(GRIP-pS) + PICK1 = R1(pS)R2(GRIP-pS-PICK1) | 2.8 l/(µmol*s) | 5 1/s | DBC based on Eq. 101 |
| 113 | R1R2(pS_2_-CaPICK1) + PICK1 = R1R2(pS_2_-PICK1-CaPICK1) | 2.8 l/(µmol*s) | 5 1/s | DBC based on Eq. 101 |
| 114 | R1R2(pS_2_-Ca_2_PICK1) + PICK1 = R1R2(pS_2_-PICK1-Ca_2_PICK1) | 2.8 l/(µmol*s) | 5 1/s | DBC based on Eq. 101 |
| 115 | R1(pS-SAP)R2(GRIP-pS) + PICK1 = R1(pS-SAP)R2(GRIP-pS-PICK1) | 2.8 l/(µmol*s) | 5 1/s | DBC based on Eq. 101 |
| 116 | R1(pS_2_)R2(GRIP-pS) + PICK1 = R1(pS_2_)R2(GRIP-pS-PICK1) | 2.8 l/(µmol*s) | 5 1/s | DBC based on Eq. 101 |
| 117 | R1(pS_2_-SAP)R2(GRIP-pS) + PICK1 = R1(pS_2_-SAP)R2(GRIP-pS-PICK1) | 2.8 l/(µmol*s) | 5 1/s | DBC based on Eq. 101 |
| 118 | R1(pS_2_-SAP_2_)R2(GRIP-pS) + PICK1 = R1(pS_2_-SAP_2_)R2(GRIP-pS-PICK1) | 2.8 l/(µmol*s) | 5 1/s | DBC based on Eq. 101 |
| 119 | R1R2(pS_2_) + PICK1 = R1R2(pS_2_-PICK1) | 2.8 l/(µmol*s) | 5 1/s | DBC based on Eq. 101 |
| 120 | R1R2(pS_2_-PICK1) + PICK1 = R1R2(pS_2_-PICK1-PICK1) | 2.8 l/(µmol*s) | 5 1/s | DBC based on Eq. 101 |
| 121 | R1(pS)R2(pS_2_) + PICK1 = R1(pS)R2(pS_2_-PICK1) | 2.8 l/(µmol*s) | 5 1/s | DBC based on Eq. 101 |
| 122 | PKAcAMP_4_ = R2_cAMP_4_ + 2 * PKAc | 0.0016 1/s | 170 l²/(µmol²*s) | [8,52] |
| 123 | R1R2(GRIP_2_) + PKAc = R1R2(GRIP_2_)-PKAc | 4.02 l/(µmol*s) | 24 1/s | [19,20] |
| 124 | R1R2(pS_2_) + PKAc = R1R2(pS_2_)-PKAc | 4.02 l/(µmol*s) | 24 1/s | DBC based on Eq. 123 |
| 125 | R1R2(pS_2_-PICK1) + PKAc = R1R2(pS_2_-PICK1)-PKAc | 4.02 l/(µmol*s) | 24 1/s | DBC based on Eq. 123 |
| 126 | R1R2(pS_2_-PICK1-PICK1) + PKAc = R1R2(pS_2_-PICK1-PICK1)-PKAc | 4.02 l/(µmol*s) | 24 1/s | DBC based on Eq. 123 |
| 127 | R1(pS)R2(pS_2_) + PKAc = R1(pS)R2(pS_2_)-PKAc | 4.02 l/(µmol*s) | 24 1/s | DBC based on Eq. 123 |
| 128 | R1(pS-SAP)R2(pS_2_) + PKAc = R1(pS-SAP)R2(pS_2_)-PKAc | 4.02 l/(µmol*s) | 24 1/s | DBC based on Eq. 123 |
| 129 | R1(pS)R2(pS_2_-PICK1) + PKAc = R1(pS)R2(pS_2_-PICK1)-PKAc | 4.02 l/(µmol*s) | 24 1/s | DBC based on Eq. 123 |
| 130 | R1(pS-SAP)R2(pS_2_-PICK1) + PKAc = R1(pS-SAP)R2(pS_2_-PICK1)-PKAc | 4.02 l/(µmol*s) | 24 1/s | DBC based on Eq. 123 |
| 131 | R1(pS)R2(pS_2_-PICK1-PICK1) + PKAc = R1(pS)R2(pS_2_-PICK1-PICK1)-PKAc | 4.02 l/(µmol*s) | 24 1/s | DBC based on Eq. 123 |
| 132 | R1(pS-SAP)R2(pS_2_-PICK1-PICK1) + PKAc = R1(pS-SAP)R2(pS_2_-PICK1-PICK1)-PKAc | 4.02 l/(µmol*s) | 24 1/s | DBC based on Eq. 123 |
| 133 | R1(pS)R2(GRIP_2_) + PKAc = R1(pS)R2(GRIP_2_)-PKAc | 4.02 l/(µmol*s) | 24 1/s | DBC based on Eq. 123 |
| 134 | R1(pS-SAP)R2(GRIP_2_) + PKAc = R1(pS-SAP)R2(GRIP_2_)-PKAc | 4.02 l/(µmol*s) | 24 1/s | DBC based on Eq. 123 |
| 135 | R1R2(GRIP-pS) + PKAc = R1R2(GRIP-pS)-PKAc | 4.02 l/(µmol*s) | 24 1/s | DBC based on Eq. 123 |
| 136 | R1R2(GRIP-pS-PICK1) + PKAc = R1R2(GRIP-pS-PICK1)-PKAc | 4.02 l/(µmol*s) | 24 1/s | DBC based on Eq. 123 |
| 137 | R1(pS)R2(GRIP-pS) + PKAc = R1(pS)R2(GRIP-pS)-PKAc | 4.02 l/(µmol*s) | 24 1/s | DBC based on Eq. 123 |
| 138 | R1(pS-SAP)R2(GRIP-pS) + PKAc = R1(pS-SAP)R2(GRIP-pS)-PKAc | 4.02 l/(µmol*s) | 24 1/s | DBC based on Eq. 123 |
| 139 | R1(pS)R2(GRIP-pS-PICK1) + PKAc = R1(pS)R2(GRIP-pS-PICK1)-PKAc | 4.02 l/(µmol*s) | 24 1/s | DBC based on Eq. 123 |
| 140 | R1(pS-SAP)R2(GRIP-pS-PICK1) + PKAc = R1(pS-SAP)R2(GRIP-pS-PICK1)-PKAc | 4.02 l/(µmol*s) | 24 1/s | DBC based on Eq. 123 |
| 141 | PKA + 2 * cAMP = PKAcAMP_2_ | 87 l²/(µmol²*s) | 0.02 1/s | [8,53,54] |
| 142 | PKAcAMP_2_ + 2 * cAMP = PKAcAMP_4_ | 115.4 l²/(µmol²*s) | 0.2 1/s | [8,53,54] |
| 143 | R1R2(GRIP_2_) + Ca_3_PKC = R1R2(GRIP_2_)-Ca_3_PKC | 0.8 l/(µmol*s) | 0.8 1/s | [7] |
| 144 | R1(pS-SAP)R2(GRIP-pS) + Ca_3_PKC = R1(pS-SAP)R2(GRIP-pS)-Ca_3_PKC | 0.8 l/(µmol*s) | 0.8 1/s | DBC based on Eq. 143 |
| 145 | R1(pS)R2(GRIP-pS-PICK1) + Ca_3_PKC = R1(pS)R2(GRIP-pS-PICK1)-Ca_3_PKC | 0.8 l/(µmol*s) | 0.8 1/s | DBC based on Eq. 143 |
| 146 | R1(pS-SAP)R2(GRIP-pS-PICK1) + Ca_3_PKC = R1(pS-SAP)R2(GRIP-pS-PICK1)-Ca_3_PKC | 0.8 l/(µmol*s) | 0.8 1/s | DBC based on Eq. 143 |
| 147 | R1(pS_2_)R2(GRIP-pS) + Ca_3_PKC = R1(pS_2_)R2(GRIP-pS)-Ca_3_PKC | 0.8 l/(µmol*s) | 0.8 1/s | DBC based on Eq. 143 |
| 148 | R1(pS_2_-SAP)R2(GRIP-pS) + Ca_3_PKC = R1(pS_2_-SAP)R2(GRIP-pS)-Ca_3_PKC | 0.8 l/(µmol*s) | 0.8 1/s | DBC based on Eq. 143 |
| 149 | R1(pS_2_-SAP_2_)R2(GRIP-pS) + Ca_3_PKC = R1(pS_2_-SAP_2_)R2(GRIP-pS)-Ca_3_PKC | 0.8 l/(µmol*s) | 0.8 1/s | DBC based on Eq. 143 |
| 150 | R1(pS_2_)R2(GRIP-pS-PICK1) + Ca_3_PKC = R1(pS_2_)R2(GRIP-pS-PICK1)-Ca_3_PKC | 0.8 l/(µmol*s) | 0.8 1/s | DBC based on Eq. 143 |
| 151 | R1(pS_2_-SAP)R2(GRIP-pS-PICK1) + Ca_3_PKC = R1(pS_2_-SAP)R2(GRIP-pS-PICK1)-Ca_3_PKC | 0.8 l/(µmol*s) | 0.8 1/s | DBC based on Eq. 143 |
| 152 | R1(pS_2_-SAP_2_)R2(GRIP-pS-PICK1) + Ca_3_PKC = R1(pS_2_-SAP_2_)R2(GRIP-pS-PICK1)-Ca_3_PKC | 0.8 l/(µmol*s) | 0.8 1/s | DBC based on Eq. 143 |
| 153 | R1(pS)R2(GRIP_2_) + Ca_3_PKC = R1(pS)R2(GRIP_2_)-Ca_3_PKC | 0.8 l/(µmol*s) | 0.8 1/s | DBC based on Eq. 143 |
| 154 | R1(pS-SAP)R2(GRIP_2_) + Ca_3_PKC = R1(pS-SAP)R2(GRIP_2_)-Ca_3_PKC | 0.8 l/(µmol*s) | 0.8 1/s | DBC based on Eq. 143 |
| 155 | R1(pS_2_)R2(GRIP_2_) + Ca_3_PKC = R1(pS_2_)R2(GRIP_2_)-Ca_3_PKC | 0.8 l/(µmol*s) | 0.8 1/s | DBC based on Eq. 143 |
| 156 | R1(pS_2_-SAP)R2(GRIP_2_) + Ca_3_PKC = R1(pS_2_-SAP)R2(GRIP_2_)-Ca_3_PKC | 0.8 l/(µmol*s) | 0.8 1/s | DBC based on Eq. 143 |
| 157 | R1(pS_2_-SAP_2_)R2(GRIP_2_) + Ca_3_PKC = R1(pS_2_-SAP_2_)R2(GRIP_2_)-Ca_3_PKC | 0.8 l/(µmol*s) | 0.8 1/s | DBC based on Eq. 143 |
| 158 | R1R2(GRIP-pS) + Ca_3_PKC = R1R2(GRIP-pS)-Ca_3_PKC | 0.8 l/(µmol*s) | 0.8 1/s | DBC based on Eq. 143 |
| 159 | R1R2(GRIP-pS-PICK1) + Ca_3_PKC = R1R2(GRIP-pS-PICK1)-Ca_3_PKC | 0.8 l/(µmol*s) | 0.8 1/s | DBC based on Eq. 143 |
| 160 | R1(pS)R2(GRIP-pS) + Ca_3_PKC = R1(pS)R2(GRIP-pS)-Ca_3_PKC | 0.8 l/(µmol*s) | 0.8 1/s | DBC based on Eq. 143 |
| 161 | Ca + PMCA = Ca_PMCA | 2500 l/(µmol*s) | 2000 1/s | [6,7] |
| 162 | Ca_PMCA -> PMCA | 125 1/s |  | [6,7] |
| 163 | R1(pS)R2(GRIP_2_) + PP2B-CaMCa_4_ = R1(pS)R2(GRIP_2_)-PP2B-CaMCa_4_ | 2.01 l/(µmol*s) | 8 1/s | [19,20] |
| 164 | R1(pS_2_)R2(pS_2_) + PP2B-CaMCa_4_ = R1(pS_2_)R2(pS_2_)-PP2B-CaMCa_4_ | 2.01 l/(µmol*s) | 8 1/s | DBC based on Eq. 163 |
| 165 | R1(pS_2_-SAP)R2(pS_2_) + PP2B-CaMCa_4_ = R1(pS_2_-SAP)R2(pS_2_)-PP2B-CaMCa_4_ | 2.01 l/(µmol*s) | 8 1/s | DBC based on Eq. 163 |
| 166 | R1(pS_2_)R2(pS_2_-PICK1) + PP2B-CaMCa_4_ = R1(pS_2_)R2(pS_2_-PICK1)-PP2B-CaMCa_4_ | 2.01 l/(µmol*s) | 8 1/s | DBC based on Eq. 163 |
| 167 | R1(pS_2_-SAP)R2(pS_2_-PICK1) + PP2B-CaMCa_4_ = R1(pS_2_-SAP)R2(pS_2_-PICK1)-PP2B-CaMCa_4_ | 2.01 l/(µmol*s) | 8 1/s | DBC based on Eq. 163 |
| 168 | R1(pS_2_)R2(pS_2_-PICK1-PICK1) + PP2B-CaMCa_4_ = R1(pS_2_)R2(pS_2_-PICK1-PICK1)-PP2B-CaMCa_4_ | 2.01 l/(µmol*s) | 8 1/s | DBC based on Eq. 163 |
| 169 | R1(pS_2_-SAP)R2(pS_2_-PICK1-PICK1) + PP2B-CaMCa_4_ = R1(pS_2_-SAP)R2(pS_2_-PICK1-PICK1)-PP2B-CaMCa_4_ | 2.01 l/(µmol*s) | 8 1/s | DBC based on Eq. 163 |
| 170 | R1(pS)R2(pS_2_) + PP2B-CaMCa_4_ = R1(pS)R2(pS_2_)-PP2B-CaMCa_4_ | 2.01 l/(µmol*s) | 8 1/s | DBC based on Eq. 163 |
| 171 | R1(pS)R2(pS_2_-PICK1) + PP2B-CaMCa_4_ = R1(pS)R2(pS_2_-PICK1)-PP2B-CaMCa_4_ | 2.01 l/(µmol*s) | 8 1/s | DBC based on Eq. 163 |
| 172 | R1(pS)R2(pS_2_-PICK1-PICK1) + PP2B-CaMCa_4_ = R1(pS)R2(pS_2_-PICK1-PICK1)-PP2B-CaMCa_4_ | 2.01 l/(µmol*s) | 8 1/s | DBC based on Eq. 163 |
| 173 | R1(pS_2_)R2(GRIP_2_) + PP2B-CaMCa_4_ = R1(pS_2_)R2(GRIP_2_)-PP2B-CaMCa_4_ | 2.01 l/(µmol*s) | 8 1/s | DBC based on Eq. 163 |
| 174 | R1(pS_2_-SAP)R2(GRIP_2_) + PP2B-CaMCa_4_ = R1(pS_2_-SAP)R2(GRIP_2_)-PP2B-CaMCa_4_ | 2.01 l/(µmol*s) | 8 1/s | DBC based on Eq. 163 |
| 175 | R1(pS)R2(GRIP-pS) + PP2B-CaMCa_4_ = R1(pS)R2(GRIP-pS)-PP2B-CaMCa_4_ | 2.01 l/(µmol*s) | 8 1/s | DBC based on Eq. 163 |
| 176 | R1(pS)R2(GRIP-pS-PICK1) + PP2B-CaMCa_4_ = R1(pS)R2(GRIP-pS-PICK1)-PP2B-CaMCa_4_ | 2.01 l/(µmol*s) | 8 1/s | DBC based on Eq. 163 |
| 177 | R1(pS_2_)R2(GRIP-pS) + PP2B-CaMCa_4_ = R1(pS_2_)R2(GRIP-pS)-PP2B-CaMCa_4_ | 2.01 l/(µmol*s) | 8 1/s | DBC based on Eq. 163 |
| 178 | R1(pS_2_-SAP)R2(GRIP-pS) + PP2B-CaMCa_4_ = R1(pS_2_-SAP)R2(GRIP-pS)-PP2B-CaMCa_4_ | 2.01 l/(µmol*s) | 8 1/s | DBC based on Eq. 163 |
| 179 | R1(pS_2_)R2(GRIP-pS-PICK1) + PP2B-CaMCa_4_ = R1(pS_2_)R2(GRIP-pS-PICK1)-PP2B-CaMCa_4_ | 2.01 l/(µmol*s) | 8 1/s | DBC based on Eq. 163 |
| 180 | R1(pS_2_-SAP)R2(GRIP-pS-PICK1) + PP2B-CaMCa_4_ = R1(pS_2_-SAP)R2(GRIP-pS-PICK1)-PP2B-CaMCa_4_ | 2.01 l/(µmol*s) | 8 1/s | DBC based on Eq. 163 |
| 181 | PP2B + CaM = PP2B-CaM | 4.6 l/(µmol*s) | 0.0012 1/s | DBC, [20] |
| 182 | PP2B + CaMCa_2_ = PP2B-CaMCa_2_ | 46 l/(µmol*s) | 0.0012 1/s | [20,22] |
| 183 | PP2B-CaM + 2 * Ca = PP2B-CaMCa_2_ | 6000 l²/(µmol²*s) | 0.91 1/s | [20,23] |
| 184 | PP2B + CaMCa_4_ = PP2B-CaMCa_4_ | 46 l/(µmol*s) | 0.0012 1/s | [20,22] |
| 185 | PP2B-CaMCa_2_ + 2 * Ca = PP2B-CaMCa_4_ | 100000 l²/(µmol²*s) | 1000 1/s | DBC, [20] |
| 186 | PDE4-PKAc -> pPDE4 + PKAc | 0.125 1/s |  | [8,9,13] |
| 187 | pPDE4 -> PDE4 | 0.0025 1/s |  | [8] |
| 188 | pPDE4 + cAMP = pPDE4-cAMP | 43.32 l/(µmol*s) | 137.9 1/s | [8,9,13] |
| 189 | PDE4-cAMP-PKAc -> pPDE4-cAMP + PKAc | 0.125 1/s |  | [8,9,13] |
| 190 | R1(pS)R2(GRIP_2_)-PP2B-CaMCa_4_ -> R1R2(GRIP_2_) + PP2B-CaMCa_4_ | 2 1/s |  | [19,20] |
| 191 | R1(pS_2_)R2(pS_2_)-PP2B-CaMCa_4_ -> R1(pS)R2(pS_2_) + PP2B-CaMCa_4_ | 2 1/s |  | [19,20] |
| 192 | R1(pS_2_-SAP)R2(pS_2_)-PP2B-CaMCa_4_ -> R1(pS-SAP)R2(pS_2_) + PP2B-CaMCa_4_ | 2 1/s |  | [19,20] |
| 193 | R1(pS_2_)R2(pS_2_-PICK1)-PP2B-CaMCa_4_ -> R1(pS)R2(pS_2_-PICK1) + PP2B-CaMCa_4_ | 2 1/s |  | [19,20] |
| 194 | R1(pS_2_-SAP)R2(pS_2_-PICK1)-PP2B-CaMCa_4_ -> R1(pS-SAP)R2(pS_2_-PICK1) + PP2B-CaMCa_4_ | 2 1/s |  | [19,20] |
| 195 | R1(pS_2_)R2(pS_2_-PICK1-PICK1)-PP2B-CaMCa_4_ -> R1(pS)R2(pS_2_-PICK1-PICK1) + PP2B-CaMCa_4_ | 2 1/s |  | [19,20] |
| 196 | R1(pS_2_-SAP)R2(pS_2_-PICK1-PICK1)-PP2B-CaMCa_4_ -> R1(pS-SAP)R2(pS_2_-PICK1-PICK1) + PP2B-CaMCa_4_ | 2 1/s |  | [19,20] |
| 197 | R1(pS)R2(pS_2_)-PP2B-CaMCa_4_ -> R1R2(pS_2_) + PP2B-CaMCa_4_ | 2 1/s |  | [19,20] |
| 198 | R1(pS)R2(pS_2_-PICK1)-PP2B-CaMCa_4_ -> R1R2(pS_2_-PICK1) + PP2B-CaMCa_4_ | 2 1/s |  | [19,20] |
| 199 | R1(pS)R2(pS_2_-PICK1-PICK1)-PP2B-CaMCa_4_ -> R1R2(pS_2_-PICK1-PICK1) + PP2B-CaMCa_4_ | 2 1/s |  | [19,20] |
| 200 | R1(pS_2_)R2(GRIP_2_)-PP2B-CaMCa_4_ -> R1(pS)R2(GRIP_2_) + PP2B-CaMCa_4_ | 2 1/s |  | [19,20] |
| 201 | R1(pS_2_-SAP)R2(GRIP_2_)-PP2B-CaMCa_4_ -> R1(pS-SAP)R2(GRIP_2_) + PP2B-CaMCa_4_ | 2 1/s |  | [19,20] |
| 202 | R1(pS)R2(GRIP-pS)-PP2B-CaMCa_4_ -> R1R2(GRIP-pS) + PP2B-CaMCa_4_ | 2 1/s |  | [19,20] |
| 203 | R1(pS)R2(GRIP-pS-PICK1)-PP2B-CaMCa_4_ -> R1R2(GRIP-pS-PICK1) + PP2B-CaMCa_4_ | 2 1/s |  | [19,20] |
| 204 | R1(pS_2_)R2(GRIP-pS)-PP2B-CaMCa_4_ -> R1(pS)R2(GRIP-pS) + PP2B-CaMCa_4_ | 2 1/s |  | [19,20] |
| 205 | R1(pS_2_-SAP)R2(GRIP-pS)-PP2B-CaMCa_4_ -> R1(pS-SAP)R2(GRIP-pS) + PP2B-CaMCa_4_ | 2 1/s |  | [19,20] |
| 206 | R1(pS_2_)R2(GRIP-pS-PICK1)-PP2B-CaMCa_4_ -> R1(pS)R2(GRIP-pS-PICK1) + PP2B-CaMCa_4_ | 2 1/s |  | [19,20] |
| 207 | R1(pS_2_-SAP)R2(GRIP-pS-PICK1)-PP2B-CaMCa_4_ -> R1(pS-SAP)R2(GRIP-pS-PICK1) + PP2B-CaMCa_4_ | 2 1/s |  | [19,20] |
| 208 | R1R2(GRIP_2_)-PKAc -> R1(pS)R2(GRIP_2_) + PKAc | 6 1/s |  | [19,20] |
| 209 | R1R2(pS_2_)-PKAc -> R1(pS)R2(pS_2_) + PKAc | 6 1/s |  | [19,20] |
| 210 | R1R2(pS_2_-PICK1)-PKAc -> R1(pS)R2(pS_2_-PICK1) + PKAc | 6 1/s |  | [19,20] |
| 211 | R1R2(pS_2_-PICK1-PICK1)-PKAc -> R1(pS)R2(pS_2_-PICK1-PICK1) + PKAc | 6 1/s |  | [19,20] |
| 212 | R1(pS)R2(pS_2_)-PKAc -> R1(pS_2_)R2(pS_2_) + PKAc | 6 1/s |  | [19,20] |
| 213 | R1(pS-SAP)R2(pS_2_)-PKAc -> R1(pS_2_-SAP)R2(pS_2_) + PKAc | 6 1/s |  | [19,20] |
| 214 | R1(pS)R2(pS_2_-PICK1)-PKAc -> R1(pS_2_)R2(pS_2_-PICK1) + PKAc | 6 1/s |  | [19,20] |
| 215 | R1(pS-SAP)R2(pS_2_-PICK1)-PKAc -> R1(pS_2_-SAP)R2(pS_2_-PICK1) + PKAc | 6 1/s |  | [19,20] |
| 216 | R1(pS)R2(pS_2_-PICK1-PICK1)-PKAc -> R1(pS_2_)R2(pS_2_-PICK1-PICK1) + PKAc | 6 1/s |  | [19,20] |
| 217 | R1(pS-SAP)R2(pS_2_-PICK1-PICK1)-PKAc -> R1(pS_2_-SAP)R2(pS_2_-PICK1-PICK1) + PKAc | 6 1/s | ﻿ | [19,20] |
| 218 | R1(pS)R2(GRIP_2_)-PKAc -> R1(pS_2_)R2(GRIP_2_) + PKAc | 6 1/s |  | [19,20] |
| 219 | R1(pS-SAP)R2(GRIP_2_)-PKAc -> R1(pS_2_-SAP)R2(GRIP_2_) + PKAc | 6 1/s |  | [19,20] |
| 220 | R1R2(GRIP-pS)-PKAc -> R1(pS)R2(GRIP-pS) + PKAc | 6 1/s |  | [19,20] |
| 221 | R1R2(GRIP-pS-PICK1)-PKAc -> R1(pS)R2(GRIP-pS-PICK1) + PKAc | 6 1/s |  | [19,20] |
| 222 | R1(pS)R2(GRIP-pS)-PKAc -> R1(pS_2_)R2(GRIP-pS) + PKAc | 6 1/s |  | [19,20] |
| 223 | R1(pS-SAP)R2(GRIP-pS)-PKAc -> R1(pS_2_-SAP)R2(GRIP-pS) + PKAc | 6 1/s |  | [19,20] |
| 224 | R1(pS)R2(GRIP-pS-PICK1)-PKAc -> R1(pS_2_)R2(GRIP-pS-PICK1) + PKAc | 6 1/s |  | [19,20] |
| 225 | R1(pS-SAP)R2(GRIP-pS-PICK1)-PKAc -> R1(pS_2_-SAP)R2(GRIP-pS-PICK1) + PKAc | 6 1/s |  | [19,20] |
| 226 | R1R2(GRIP_2_)-Ca_3_PKC -> R1R2(GRIP-pS) + GRIP + Ca_3_PKC | 5 1/s |  | [7] |
| 227 | R1(pS-SAP)R2(GRIP-pS)-Ca_3_PKC -> R1(pS-SAP)R2(pS_2_) + GRIP + Ca_3_PKC | 5 1/s |  | [7] |
| 228 | R1(pS)R2(GRIP-pS-PICK1)-Ca_3_PKC -> R1(pS)R2(pS_2_-PICK1) + GRIP + Ca_3_PKC | 5 1/s |  | [7] |
| 229 | R1(pS-SAP)R2(GRIP-pS-PICK1)-Ca_3_PKC -> R1(pS-SAP)R2(pS_2_-PICK1) + GRIP + Ca_3_PKC | 5 1/s |  | [7] |
| 230 | R1(pS_2_)R2(GRIP-pS)-Ca_3_PKC -> R1(pS_2_)R2(pS_2_) + GRIP + Ca_3_PKC | 5 1/s |  | [7] |
| 231 | R1(pS_2_-SAP)R2(GRIP-pS)-Ca_3_PKC -> R1(pS_2_-SAP)R2(pS_2_) + GRIP + Ca_3_PKC | 5 1/s |  | [7] |
| 232 | R1(pS_2_-SAP_2_)R2(GRIP-pS)-Ca_3_PKC -> R1(pS_2_-SAP_2_)R2(pS_2_) + GRIP + Ca_3_PKC | 5 1/s |  | [7] |
| 233 | R1(pS_2_)R2(GRIP-pS-PICK1)-Ca_3_PKC -> R1(pS_2_)R2(pS_2_-PICK1) + GRIP + Ca_3_PKC | 5 1/s |  | [7] |
| 234 | R1(pS_2_-SAP)R2(GRIP-pS-PICK1)-Ca_3_PKC -> R1(pS_2_-SAP)R2(pS_2_-PICK1) + GRIP + Ca_3_PKC | 5 1/s |  | [7] |
| 235 | R1(pS_2_-SAP_2_)R2(GRIP-pS-PICK1)-Ca_3_PKC -> R1(pS_2_-SAP_2_)R2(pS_2_-PICK1) + GRIP + Ca_3_PKC | 5 1/s |  | [7] |
| 236 | R1(pS)R2(GRIP_2_)-Ca_3_PKC -> R1(pS)R2(GRIP-pS) + GRIP + Ca_3_PKC | 5 1/s |  | [7] |
| 237 | R1(pS-SAP)R2(GRIP_2_)-Ca_3_PKC -> R1(pS-SAP)R2(GRIP-pS) + GRIP + Ca_3_PKC | 5 1/s |  | [7] |
| 238 | R1(pS_2_)R2(GRIP_2_)-Ca_3_PKC -> R1(pS_2_)R2(GRIP-pS) + GRIP + Ca_3_PKC | 5 1/s |  | [7] |
| 239 | R1(pS_2_-SAP)R2(GRIP_2_)-Ca_3_PKC -> R1(pS_2_-SAP)R2(GRIP-pS) + GRIP + Ca_3_PKC | 5 1/s |  | [7] |
| 240 | R1(pS_2_-SAP_2_)R2(GRIP_2_)-Ca_3_PKC -> R1(pS_2_-SAP_2_)R2(GRIP-pS) + GRIP + Ca_3_PKC | 5 1/s |  | [7] |
| 241 | R1R2(GRIP-pS)-Ca_3_PKC -> R1R2(pS_2_) + GRIP + Ca_3_PKC | 5 1/s |  | [7] |
| 242 | R1R2(GRIP-pS-PICK1)-Ca_3_PKC -> R1R2(pS_2_-PICK1) + GRIP + Ca_3_PKC | 5 1/s |  | [7] |
| 243 | R1(pS)R2(GRIP-pS)-Ca_3_PKC -> R1(pS)R2(pS_2_) + GRIP + Ca_3_PKC | 5 1/s |  | [7] |
| 244 | R1R2endo(GRIP_2_)MyoV -> R1R2psd(GRIP_2_)MyoV | 0.02 1/s |  | This work based on Ref. [37] |
| 245 | R1R2endo(GRIP_2_) + MyoV -> R1R2endo(GRIP_2_)MyoV | 0.001 l/(µmol*s) |  | This work |
| 246 | R1R2psd(GRIP_2_)MyoVSyt1 -> R1R2(GRIP_2_) + MyoV + Syt1 | 2.5e-05 1/s |  | This work |
| 247 | R1R2psd(GRIP_2_)MyoV(Ca)_A_Syt1 -> R1R2(GRIP_2_) + MyoV + (Ca)_A_Syt1 | 0.0001 1/s |  | This work |
| 248 | R1R2psd(GRIP_2_)MyoV(Ca_2_)_A_Syt1 -> R1R2(GRIP_2_) + MyoV + (Ca_2_)_A_Syt1 | 0.3 1/s |  | This work |
| 249 | R1R2psd(GRIP_2_)MyoV(Ca_2_)_A_(Ca)_B_Syt1 -> R1R2(GRIP_2_) + MyoV + (Ca_2_)_A_(Ca)_B_Syt1 | 20 1/s |  | This work |
| 250 | R1R2psd(GRIP_2_)MyoV(Ca_2_)_A_(Ca_2_)_B_Syt1 -> R1R2(GRIP_2_) + MyoV + (Ca_2_)_A_(Ca_2_)_B_Syt1 | 60 1/s |  | This work |
| 251 | R1R2psd(GRIP_2_)MyoV(Ca_3_)_A_(Ca_2_)_B_Syt1 -> R1R2(GRIP_2_) + MyoV + (Ca_3_)_A_(Ca_2_)_B_Syt1 | 70 1/s |  | This work |
| 252 | R1R2psd(GRIP_2_)MyoV + (Ca)_A_Syt1 -> R1R2psd(GRIP_2_)MyoV(Ca)_A_Syt1 | 10000 l/(µmol*s) |  | An approximation treatment for Syt1 introduced in this work (see the SI text) |
| 253 | R1R2psd(GRIP_2_)MyoVSyt1 + Ca = R1R2psd(GRIP_2_)MyoV(Ca)_A_Syt1 | 2 l/(µmol*s) | 97.6 1/s | DBC based on Eq. 1 |
| 254 | R1R2psd(GRIP_2_)MyoV + (Ca_2_)_A_(Ca)_B_Syt1 -> R1R2psd(GRIP_2_)MyoV(Ca_2_)_A_(Ca)_B_Syt1 | 10000 l/(µmol*s) |  | An approximation treatment for Syt1 introduced in this work (see the SI text) |
| 255 | R1R2psd(GRIP_2_)MyoV(Ca_2_)_A_Syt1 + Ca = R1R2psd(GRIP_2_)MyoV(Ca_2_)_A_(Ca)_B_Syt1 | 1 l/(µmol*s) | 142 1/s | DBC based on Eq. 2 |
| 256 | R1R2psd(GRIP_2_)MyoV + (Ca_2_)_A_(Ca_2_)_B_Syt1 -> R1R2psd(GRIP_2_)MyoV(Ca_2_)_A_(Ca_2_)_B_Syt1 | 10000 l/(µmol*s) |  | An approximation treatment for Syt1 introduced in this work (see the SI text) |
| 257 | R1R2psd(GRIP_2_)MyoV(Ca_2_)_A_(Ca)_B_Syt1 + Ca = R1R2psd(GRIP_2_)MyoV(Ca_2_)_A_(Ca_2_)_B_Syt1 | 1 l/(µmol*s) | 142 1/s | DBC based on Eq. 3 |
| 258 | R1R2psd(GRIP_2_)MyoV + (Ca_2_)_A_Syt1 -> R1R2psd(GRIP_2_)MyoV(Ca_2_)_A_Syt1 | 10000 l/(µmol*s) |  | An approximation treatment for Syt1 introduced in this work (see the SI text) |
| 259 | R1R2psd(GRIP_2_)MyoV(Ca)_A_Syt1 + Ca = R1R2psd(GRIP_2_)MyoV(Ca_2_)_A_Syt1 | 1 l/(µmol*s) | 488 1/s | DBC based on Eq. 4 |
| 260 | R1R2psd(GRIP_2_)MyoV + (Ca_3_)_A_(Ca_2_)_B_Syt1 -> R1R2psd(GRIP_2_)MyoV(Ca_3_)_A_(Ca_2_)_B_Syt1 | 10000 l/(µmol*s) |  | An approximation treatment for Syt1 introduced in this work (see the SI text) |
| 261 | R1R2psd(GRIP_2_)MyoV(Ca_2_)_A_(Ca_2_)_B_Syt1 + Ca = R1R2psd(GRIP_2_)MyoV(Ca_3_)_A_(Ca_2_)_B_Syt1 | 0.5 l/(µmol*s) | 1560 1/s | DBC based on Eq. 5 |
| 262 | R1R2psd(GRIP_2_)MyoV + Syt1 -> R1R2psd(GRIP_2_)MyoVSyt1 | 10000 l/(µmol*s) |  | An approximation treatment for Syt1 introduced in this work (see the SI text) |
| 263 | R2 + 2 * PKAc -> PKA | 0.1 l²/(µmol²*s) |  | This work |
| 264 | R2_cAMP_4_ = R2 + 4 * cAMP | 1 1/s | 10000 l^4/(µmol^4*s) | [21] |
| 265 | R1(pS)R2(GRIP_2_) + SAP = R1(pS-SAP)R2(GRIP_2_) | 1.5 l/(µmol*s) | 1 1/s | This work |
| 266 | R1(pS)R2(pS_2_) + SAP = R1(pS-SAP)R2(pS_2_) | 1.5 l/(µmol*s) | 1 1/s | DBC based on Eq. 265 |
| 267 | R1(pS)R2(pS_2_-PICK1) + SAP = R1(pS-SAP)R2(pS_2_-PICK1) | 1.5 l/(µmol*s) | 1 1/s | DBC based on Eq. 265 |
| 268 | R1(pS)R2(pS_2_-PICK1-PICK1) + SAP = R1(pS-SAP)R2(pS_2_-PICK1-PICK1) | 1.5 l/(µmol*s) | 1 1/s | DBC based on Eq. 265 |
| 269 | R1(pS_2_)R2(pS_2_) + SAP = R1(pS_2_-SAP)R2(pS_2_) | 1.5 l/(µmol*s) | 1 1/s | DBC based on Eq. 265 |
| 270 | R1(pS_2_-SAP)R2(pS_2_) + SAP = R1(pS_2_-SAP_2_)R2(pS_2_) | 1.5 l/(µmol*s) | 1 1/s | DBC based on Eq. 265 |
| 271 | R1(pS_2_)R2(pS_2_-PICK1) + SAP = R1(pS_2_-SAP)R2(pS_2_-PICK1) | 1.5 l/(µmol*s) | 1 1/s | DBC based on Eq. 265 |
| 272 | R1(pS_2_-SAP)R2(pS_2_-PICK1) + SAP = R1(pS_2_-SAP_2_)R2(pS_2_-PICK1) | 1.5 l/(µmol*s) | 1 1/s | DBC based on Eq. 265 |
| 273 | R1(pS_2_)R2(pS_2_-PICK1-PICK1) + SAP = R1(pS_2_-SAP)R2(pS_2_-PICK1-PICK1) | 1.5 l/(µmol*s) | 1 1/s | DBC based on Eq. 265 |
| 274 | R1(pS_2_-SAP)R2(pS_2_-PICK1-PICK1) + SAP = R1(pS_2_-SAP_2_)R2(pS_2_-PICK1-PICK1) | 1.5 l/(µmol*s) | 1 1/s | DBC based on Eq. 265 |
| 275 | R1(pS_2_)R2(GRIP_2_) + SAP = R1(pS_2_-SAP)R2(GRIP_2_) | 1.5 l/(µmol*s) | 1 1/s | DBC based on Eq. 265 |
| 276 | R1(pS_2_-SAP)R2(GRIP_2_) + SAP = R1(pS_2_-SAP_2_)R2(GRIP_2_) | 1.5 l/(µmol*s) | 1 1/s | DBC based on Eq. 265 |
| 277 | R1(pS)R2(GRIP-pS) + SAP = R1(pS-SAP)R2(GRIP-pS) | 1.5 l/(µmol*s) | 1 1/s | DBC based on Eq. 265 |
| 278 | R1(pS)R2(GRIP-pS-PICK1) + SAP = R1(pS-SAP)R2(GRIP-pS-PICK1) | 1.5 l/(µmol*s) | 1 1/s | DBC based on Eq. 265 |
| 279 | R1(pS_2_)R2(GRIP-pS) + SAP = R1(pS_2_-SAP)R2(GRIP-pS) | 1.5 l/(µmol*s) | 1 1/s | DBC based on Eq. 265 |
| 280 | R1(pS_2_-SAP)R2(GRIP-pS) + SAP = R1(pS_2_-SAP_2_)R2(GRIP-pS) | 1.5 l/(µmol*s) | 1 1/s | DBC based on Eq. 265 |
| 281 | R1(pS_2_)R2(GRIP-pS-PICK1) + SAP = R1(pS_2_-SAP)R2(GRIP-pS-PICK1) | 1.5 l/(µmol*s) | 1 1/s | DBC based on Eq. 265 |
| 282 | R1(pS_2_-SAP)R2(GRIP-pS-PICK1) + SAP = R1(pS_2_-SAP_2_)R2(GRIP-pS-PICK1) | 1.5 l/(µmol*s) | 1 1/s | DBC based on Eq. 265 |
| 283 | Ca + SERCA = Ca_SERCA | 17147 l/(µmol*s) | 8426.3 1/s | [6,7] |
| 284 | Ca + Ca_SERCA = Ca_2__SERCA | 17147 l/(µmol*s) | 8426.3 1/s | [6,7] |
| 285 | Ca_2__SERCA -> SERCA | 250 1/s |  | [6,7] |

**Table S3. Parameters for PICK1-KO mice** [1]. The concentration of AMPAR (R1R2(GRIP_2_)) was determined so that the total concentration of AMPARs at the cytosol is equivalent to that for the wild-type model, where we assumed that a kind of regulatory mechanisms would work to maintain the concentration of AMPARs at the cytosol.

|  | **Volume (l)** | **Ref.** |
| --- | --- | --- |
| **Species** | **Initial concentration (µmol/l)** |  |
| R1R2(GRIP_2_) | 4 | This work |
| PICK1 | 0.51 | This work |

**Table S4. Parameters for LTP blockage by chemical generic inhibition of myosin V_b_ motility** [37]**.** The concentration of AMPAR (R1R2(GRIP_2_)) was determined so that the total concentration of AMPARs at the cytosol is equivalent to that for the wild-type model, where we assumed that a kind of regulatory mechanisms would work to maintain the concentration of AMPARs at the cytosol.

|  | **Volume (l)** | **Ref.** |
| --- | --- | --- |
| **Species** | **Initial concentration (µmol/l)** |  |
| R1R2(GRIP_2_) | 1.6 | This work |

| **No.** | **Reaction** | ***k*_f_** | ***k*_b_** | **Ref.** |
| --- | --- | --- | --- | --- |
| 244 | R1R2endo(GRIP_2_)MyoV -> R1R2psd(GRIP_2_)MyoV | 0.00005 1/s |  | This work |

**Table S5. Parameters for AKAP150ΔPIX gene knock-in mouse that selectively disrupt PP2B anchoring, resulting in blockage of LTD** [2]**.** The concentration of AMPAR (R1R2(GRIP_2_)) was determined so that the total concentration of AMPARs at the cytosol is equivalent to that for the wild-type model, where we assumed that a kind of regulatory mechanisms would work to maintain the concentration of AMPARs at the cytosol.

|  | **Volume (l)** | **Ref.** |
| --- | --- | --- |
| **Species** | **Initial concentration (µmol/l)** |  |
| R1R2(GRIP_2_) | 4.2 | This work |

| **No.** | **Reaction** | ***k*_f_** | ***k*_b_** | **Ref.** |
| --- | --- | --- | --- | --- |
| 190 | R1(pS)R2(GRIP_2_)-PP2B-CaMCa_4_ -> R1R2(GRIP_2_) + PP2B-CaMCa_4_ | 0.8 1/s |  | This work |
| 191 | R1(pS_2_)R2(pS_2_)-PP2B-CaMCa_4_ -> R1(pS)R2(pS_2_) + PP2B-CaMCa_4_ | 0.8 1/s |  | This work |
| 192 | R1(pS_2_-SAP)R2(pS_2_)-PP2B-CaMCa_4_ -> R1(pS-SAP)R2(pS_2_) + PP2B-CaMCa_4_ | 0.8 1/s |  | This work |
| 193 | R1(pS_2_)R2(pS_2_-PICK1)-PP2B-CaMCa_4_ -> R1(pS)R2(pS_2_-PICK1) + PP2B-CaMCa_4_ | 0.8 1/s |  | This work |
| 194 | R1(pS_2_-SAP)R2(pS_2_-PICK1)-PP2B-CaMCa_4_ -> R1(pS-SAP)R2(pS_2_-PICK1) + PP2B-CaMCa_4_ | 0.8 1/s |  | This work |
| 195 | R1(pS_2_)R2(pS_2_-PICK1-PICK1)-PP2B-CaMCa_4_ -> R1(pS)R2(pS_2_-PICK1-PICK1) + PP2B-CaMCa_4_ | 0.8 1/s |  | This work |
| 196 | R1(pS_2_-SAP)R2(pS_2_-PICK1-PICK1)-PP2B-CaMCa_4_ -> R1(pS-SAP)R2(pS_2_-PICK1-PICK1) + PP2B-CaMCa_4_ | 0.8 1/s |  | This work |
| 197 | R1(pS)R2(pS_2_)-PP2B-CaMCa_4_ -> R1R2(pS_2_) + PP2B-CaMCa_4_ | 0.8 1/s |  | This work |
| 198 | R1(pS)R2(pS_2_-PICK1)-PP2B-CaMCa_4_ -> R1R2(pS_2_-PICK1) + PP2B-CaMCa_4_ | 0.8 1/s |  | This work |
| 199 | R1(pS)R2(pS_2_-PICK1-PICK1)-PP2B-CaMCa_4_ -> R1R2(pS_2_-PICK1-PICK1) + PP2B-CaMCa_4_ | 0.8 1/s |  | This work |
| 200 | R1(pS_2_)R2(GRIP_2_)-PP2B-CaMCa_4_ -> R1(pS)R2(GRIP_2_) + PP2B-CaMCa_4_ | 0.8 1/s |  | This work |
| 201 | R1(pS_2_-SAP)R2(GRIP_2_)-PP2B-CaMCa_4_ -> R1(pS-SAP)R2(GRIP_2_) + PP2B-CaMCa_4_ | 0.8 1/s |  | This work |
| 202 | R1(pS)R2(GRIP-pS)-PP2B-CaMCa_4_ -> R1R2(GRIP-pS) + PP2B-CaMCa_4_ | 0.8 1/s |  | This work |
| 203 | R1(pS)R2(GRIP-pS-PICK1)-PP2B-CaMCa_4_ -> R1R2(GRIP-pS-PICK1) + PP2B-CaMCa_4_ | 0.8 1/s |  | This work |
| 204 | R1(pS_2_)R2(GRIP-pS)-PP2B-CaMCa_4_ -> R1(pS)R2(GRIP-pS) + PP2B-CaMCa_4_ | 0.8 1/s |  | This work |
| 205 | R1(pS_2_-SAP)R2(GRIP-pS)-PP2B-CaMCa_4_ -> R1(pS-SAP)R2(GRIP-pS) + PP2B-CaMCa_4_ | 0.8 1/s |  | This work |
| 206 | R1(pS_2_)R2(GRIP-pS-PICK1)-PP2B-CaMCa_4_ -> R1(pS)R2(GRIP-pS-PICK1) + PP2B-CaMCa_4_ | 0.8 1/s |  | This work |
| 207 | R1(pS_2_-SAP)R2(GRIP-pS-PICK1)-PP2B-CaMCa_4_ -> R1(pS-SAP)R2(GRIP-pS-PICK1) + PP2B-CaMCa_4_ | 0.8 1/s |  | This work |

**Table S6. Parameters for calcium-binding site mutations of Syt1 in both the C2A and C2B** [4]**.**

| **No.** | **Reaction** | ***k*_f_** | ***k*_b_** | **Ref.** |
| --- | --- | --- | --- | --- |
| 1 | Syt1 + Ca = (Ca)_A_Syt1 | 0.25 l/(µmol*s) | 97.6 1/s | This work |
| 2 | (Ca_2_)_A_Syt1 + Ca = (Ca_2_)_A_(Ca)_B_Syt1 | 0.25 l/(µmol*s) | 142 1/s | This work |
| 3 | (Ca_2_)_A_(Ca)_B_Syt1 + Ca = (Ca_2_)_A_(Ca_2_)_B_Syt1 | 0.25 l/(µmol*s) | 142 1/s | This work |
| 4 | (Ca)_A_Syt1 + Ca = (Ca_2_)_A_Syt1 | 0.25 l/(µmol*s) | 488 1/s | This work |
| 5 | (Ca_2_)_A_(Ca_2_)_B_Syt1 + Ca = (Ca_3_)_A_(Ca_2_)_B_Syt1 | 0.25 l/(µmol*s) | 1560 1/s | This work |
| 252 | R1R2psd(GRIP2)MyoV + (Ca)ASyt1 -> R1R2psd(GRIP2)MyoV(Ca)ASyt1 | 1000 l/(µmol*s) |  | An approximation treatment for Syt1 introduced in this work (see the SI text) |
| 253 | R1R2psd(GRIP2)MyoVSyt1 + Ca = R1R2psd(GRIP2)MyoV(Ca)ASyt1 | 0.25 l/(µmol*s) | 97.6 1/s | DBC based on Eq. 1 |
| 254 | R1R2psd(GRIP2)MyoV + (Ca2)A(Ca)BSyt1 -> R1R2psd(GRIP2)MyoV(Ca2)A(Ca)BSyt1 | 1000 l/(µmol*s) |  | An approximation treatment for Syt1 introduced in this work (see the SI text) |
| 255 | R1R2psd(GRIP2)MyoV(Ca2)ASyt1 + Ca = R1R2psd(GRIP2)MyoV(Ca2)A(Ca)BSyt1 | 0.25 l/(µmol*s) | 142 1/s | DBC based on Eq. 2 |
| 256 | R1R2psd(GRIP2)MyoV + (Ca2)A(Ca2)BSyt1 -> R1R2psd(GRIP2)MyoV(Ca2)A(Ca2)BSyt1 | 1000 l/(µmol*s) |  | An approximation treatment for Syt1 introduced in this work (see the SI text) |
| 257 | R1R2psd(GRIP2)MyoV(Ca2)A(Ca)BSyt1 + Ca = R1R2psd(GRIP2)MyoV(Ca2)A(Ca2)BSyt1 | 0.25 l/(µmol*s) | 142 1/s | DBC based on Eq. 3 |
| 258 | R1R2psd(GRIP2)MyoV + (Ca2)ASyt1 -> R1R2psd(GRIP2)MyoV(Ca2)ASyt1 | 1000 l/(µmol*s) |  | An approximation treatment for Syt1 introduced in this work (see the SI text) |
| 259 | R1R2psd(GRIP2)MyoV(Ca)ASyt1 + Ca = R1R2psd(GRIP2)MyoV(Ca2)ASyt1 | 0.25 l/(µmol*s) | 488 1/s | DBC based on Eq. 4 |
| 260 | R1R2psd(GRIP2)MyoV + (Ca3)A(Ca2)BSyt1 -> R1R2psd(GRIP2)MyoV(Ca3)A(Ca2)BSyt1 | 10000 l/(µmol*s) |  | An approximation treatment for Syt1 introduced in this work (see the SI text) |
| 261 | R1R2psd(GRIP2)MyoV(Ca2)A(Ca2)BSyt1 + Ca = R1R2psd(GRIP2)MyoV(Ca3)A(Ca2)BSyt1 | 0.25 l/(µmol*s) | 1560 1/s | DBC based on Eq. 5 |
| 262 | R1R2psd(GRIP2)MyoV + Syt1 -> R1R2psd(GRIP2)MyoVSyt1 | 1000 l/(µmol*s) |  | An approximation treatment for Syt1 introduced in this work (see the SI text) |

**REFERENCES**

1. Volk L, Kim C-H, Takamiya K, Yu Y, Huganir RL. Developmental regulation of protein interacting with C kinase 1 (PICK1) function in hippocampal synaptic plasticity and learning. PNAS. 2010;107: 21784–21789. doi:10.1073/pnas.1016103107

2. Sanderson JL, Gorski JA, Gibson ES, Lam P, Freund RK, Chick WS, et al. AKAP150-anchored calcineurin regulates synaptic plasticity by limiting synaptic incorporation of Ca2+-permeable AMPA receptors. J Neurosci. 2012;32: 15036–15052. doi:10.1523/JNEUROSCI.3326-12.2012

3. Lee HK, Takamiya K, Han JS, Man HY, Kim CH, Rumbaugh G, et al. Phosphorylation of the AMPA receptor GluR1 subunit is required for synaptic plasticity and retention of spatial memory. Cell. 2003;112: 631–643.

4. Wu D, Bacaj T, Morishita W, Goswami D, Arendt KL, Xu W, et al. Postsynaptic synaptotagmins mediate AMPA receptor exocytosis during LTP. Nature. Nature Publishing Group; 2017;544: 316–321. doi:10.1038/nature21720

5. Doi T, Kuroda S, Michikawa T, Kawato M. Inositol 1,4,5-trisphosphate-dependent Ca2+ threshold dynamics detect spike timing in cerebellar Purkinje cells. J Neurosci. 2005;25: 950–961. doi:10.1523/JNEUROSCI.2727-04.2005

6. Antunes G, De Schutter E. A stochastic signaling network mediates the probabilistic induction of cerebellar long-term depression. J Neurosci. Society for Neuroscience; 2012;32: 9288–9300. doi:10.1523/JNEUROSCI.5976-11.2012

7. Antunes G, Roque AC, Simoes-de-Souza FM. Stochastic Induction of Long-Term Potentiation and Long-Term Depression. Sci Rep. Nature Publishing Group; 2016;6: 30899. doi:10.1038/srep30899

8. Chay A, Zamparo I, Koschinski A, Zaccolo M, Blackwell KT. Control of βAR- and N-methyl-D-aspartate (NMDA) Receptor-Dependent cAMP Dynamics in Hippocampal Neurons. PLoS Comput Biol. 2016;12: e1004735. doi:10.1371/journal.pcbi.1004735

9. Jȩdrzejewska-Szmek J, Luczak V, Abel T, Blackwell KT. β-adrenergic signaling broadly contributes to LTP induction. PLoS Comput Biol. 2017;13: e1005657. doi:10.1371/journal.pcbi.1005657

10. Sharma RK, Wang JH. Regulation of cAMP concentration by calmodulin-dependent cyclic nucleotide phosphodiesterase. Biochem Cell Biol. 1986;64: 1072–1080.

11. Sharma RK, Kalra J. Molecular interaction between cAMP and calcium in calmodulin-dependent cyclic nucleotide phosphodiesterase system. Clin Invest Med. 1994;17: 374–382.

12. Herman SB, Juilfs DM, Fauman EB, Juneau P, Menetski JP. Analysis of a mutation in phosphodiesterase type 4 that alters both inhibitor activity and nucleotide selectivity. Mol Pharmacol. 2000;57: 991–999.

13. MacKenzie SJ, Baillie GS, McPhee I, MacKenzie C, Seamons R, McSorley T, et al. Long PDE4 cAMP specific phosphodiesterases are activated by protein kinase A-mediated phosphorylation of a single serine residue in Upstream Conserved Region 1 (UCR1). Br J Pharmacol. John Wiley & Sons, Ltd (10.1111); 2002;136: 421–433. doi:10.1038/sj.bjp.0704743

14. Gold MG, Stengel F, Nygren PJ, Weisbrod CR, Bruce JE, Robinson CV, et al. Architecture and dynamics of an A-kinase anchoring protein 79 (AKAP79) signaling complex. National Academy of Sciences; 2011. pp. 6426–6431. doi:10.1073/pnas.1014400108

15. Anggono V, Huganir RL. Regulation of AMPA receptor trafficking and synaptic plasticity. Curr Opin Neurobiol. 2012;22: 461–469. doi:10.1016/j.conb.2011.12.006

16. Colledge M, Dean RA, Scott GK, Langeberg LK, Huganir RL, Scott JD. Targeting of PKA to Glutamate Receptors through a MAGUK-AKAP Complex. Neuron. 2000;27: 107–119. doi:10.1016/S0896-6273(00)00013-1

17. Sanderson JL, Gorski JA, Dell’Acqua ML. NMDA Receptor-Dependent LTD Requires Transient Synaptic Incorporation of Ca2+-Permeable AMPARs Mediated by AKAP150-Anchored PKA and Calcineurin. Neuron. 2016;89: 1000–1015. doi:10.1016/j.neuron.2016.01.043

18. Diering GH, Gustina AS, Huganir RL. PKA-GluA1 Coupling via AKAP5 Controls AMPA Receptor Phosphorylation and Cell-Surface Targeting during Bidirectional Homeostatic Plasticity. Neuron. 2014;84: 790–805. doi:10.1016/j.neuron.2014.09.024

19. Hayer A, Bhalla US. Molecular switches at the synapse emerge from receptor and kinase traffic. PLoS Comput Biol. 2005;1: 137–154. doi:10.1371/journal.pcbi.0010020

20. Oliveira RF, Kim M, Blackwell KT. Subcellular location of PKA controls striatal plasticity: stochastic simulations in spiny dendrites. PLoS Comput Biol. 2012;8: e1002383. doi:10.1371/journal.pcbi.1002383

21. Williamson T, Schwartz J-M, Kell DB, Stateva L. Deterministic mathematical models of the cAMP pathway in Saccharomyces cerevisiae. BMC Syst Biol. 2009;3: 70. doi:10.1186/1752-0509-3-70

22. Quintana AR, Wang D, Forbes JE, Waxham MN. Kinetics of calmodulin binding to calcineurin. Biochem Biophys Res Commun. 2005;334: 674–680. doi:10.1016/j.bbrc.2005.06.152

23. Lindskog M, Kim M, Wikström MA, Blackwell KT, Kotaleski JH. Transient calcium and dopamine increase PKA activity and DARPP-32 phosphorylation. PLoS Comput Biol. 2006;2: e119. doi:10.1371/journal.pcbi.0020119

24. Torrecillas A, Laynez J, Menéndez M, Corbalán-García S, Gómez-Fernández JC. Calorimetric study of the interaction of the C2 domains of classical protein kinase C isoenzymes with Ca2+ and phospholipids. Biochemistry. American Chemical Society; 2004;43: 11727–11739. doi:10.1021/bi0489659

25. Lu W, Ziff EB. PICK1 interacts with ABP/GRIP to regulate AMPA receptor trafficking. Neuron. 2005;47: 407–421. doi:10.1016/j.neuron.2005.07.006

26. Hanley JG, Henley JM. PICK1 is a calcium-sensor for NMDA-induced AMPA receptor trafficking. EMBO J. EMBO Press; 2005;24: 3266–3278. doi:10.1038/sj.emboj.7600801

27. Fiuza M, Rostosky CM, Parkinson GT, Bygrave AM, Halemani N, Baptista M, et al. PICK1 regulates AMPA receptor endocytosis via direct interactions with AP2 α-appendage and dynamin. J Cell Biol. Rockefeller University Press; 2017;216: 3323–3338. doi:10.1083/jcb.201701034

28. Shi Y, Zhang L, Yuan J, Xiao H, Yang X, Niu L. Zinc binding site in PICK1 is dominantly located at the CPC motif of its PDZ domain. J Neurochem. 2008;106: 1027–1034. doi:10.1111/j.1471-4159.2008.05434.x

29. Radhakrishnan A, Stein A, Jahn R, Fasshauer D. The Ca2+ Affinity of Synaptotagmin 1 Is Markedly Increased by a Specific Interaction of Its C2B Domain with Phosphatidylinositol 4,5-Bisphosphate. J Biol Chem. American Society for Biochemistry and Molecular Biology; 2009;284: 25749–25760. doi:10.1074/jbc.M109.042499

30. Hussain S, Egbenya DL, Lai Y-C, Dosa ZJ, Sørensen JB, Anderson AE, et al. The calcium sensor synaptotagmin 1 is expressed and regulated in hippocampal postsynaptic spines. Hippocampus. 2017;27: 1168–1177. doi:10.1002/hipo.22761

31. Jurado S, Goswami D, Zhang Y, Molina AJM, Südhof TC, Malenka RC. LTP Requires a Unique Postsynaptic SNARE Fusion Machinery. Neuron. 2013;77: 542–558. doi:10.1016/j.neuron.2012.11.029

32. Maximov A, Tang J, Yang X, Pang ZP, Südhof TC. Complexin Controls the Force Transfer from SNARE Complexes to Membranes in Fusion. Science. American Association for the Advancement of Science; 2009;323: 516–521. doi:10.1126/science.1166505

33. Südhof TC. Neurotransmitter Release: The Last Millisecond in the Life of a Synaptic Vesicle. Neuron. 2013;80: 675–690. doi:10.1016/j.neuron.2013.10.022

34. Ahmad M, Polepalli JS, Goswami D, Yang X, Kaeser-Woo YJ, Südhof TC, et al. Postsynaptic complexin controls AMPA receptor exocytosis during LTP. Neuron. 2012;73: 260–267. doi:10.1016/j.neuron.2011.11.020

35. Lu W, Shi Y, Jackson AC, Bjorgan K, During MJ, Sprengel R, et al. Subunit Composition of Synaptic AMPA Receptors Revealed by a Single-Cell Genetic Approach. Neuron. 2009;62: 254–268. doi:10.1016/j.neuron.2009.02.027

36. Mao L, Takamiya K, Thomas G, Lin D-T, Huganir RL. GRIP1 and 2 regulate activity-dependent AMPA receptor recycling via exocyst complex interactions. PNAS. 2010;107: 19038–19043. doi:10.1073/pnas.1013494107

37. Wang Z, Edwards JG, Riley N, Provance DW, Karcher R, Li X-D, et al. Myosin Vb Mobilizes Recycling Endosomes and AMPA Receptors for Postsynaptic Plasticity. Cell. 2008;135: 535–548. doi:10.1016/j.cell.2008.09.057

38. Rudolf R, Bittins CM, Gerdes H-H. The role of myosin V in exocytosis and synaptic plasticity. J Neurochem. 2011;116: 177–191. doi:10.1111/j.1471-4159.2010.07110.x

39. Kneussel M, Wagner W. Myosin motors at neuronal synapses: drivers of membrane transport and actin dynamics. Nat Rev Neurosci. Nature Publishing Group; 2013;14: 233–247. doi:10.1038/nrn3445

40. Harris KM, Stevens JK. Dendritic spines of rat cerebellar Purkinje cells: serial electron microscopy with reference to their biophysical characteristics. J Neurosci. 1988;8: 4455–4469.

41. Wang H, Storm DR. Calmodulin-regulated adenylyl cyclases: cross-talk and plasticity in the central nervous system. Mol Pharmacol. 2003;63: 463–468.

42. Sharma RK, Kalra J. Characterization of calmodulin-dependent cyclic nucleotide phosphodiesterase isoenzymes. Biochem J. Portland Press Ltd; 1994;299 ( Pt 1): 97–100.

43. Gallimore AR, Kim T, Tanaka-Yamamoto K, De Schutter E. Switching On Depression and Potentiation in the Cerebellum. Cell Rep. 2018;22: 722–733. doi:10.1016/j.celrep.2017.12.084

44. Kohout SC, Corbalán-García S, Torrecillas A, Gómez-Fernández JC, Falke JJ. C2 domains of protein kinase C isoforms alpha, beta, and gamma: activation parameters and calcium stoichiometries of the membrane-bound state. Biochemistry. 2002;41: 11411–11424.

45. Brown SE, Martin SR, Bayley PM. Kinetic control of the dissociation pathway of calmodulin-peptide complexes. J Biol Chem. 1997;272: 3389–3397.

46. Putkey JA, Kleerekoper Q, Gaertner TR, Waxham MN. A new role for IQ motif proteins in regulating calmodulin function. J Biol Chem. American Society for Biochemistry and Molecular Biology; 2003;278: 49667–49670. doi:10.1074/jbc.C300372200

47. Tang WJ, Krupinski J, Gilman AG. Expression and characterization of calmodulin-activated (type I) adenylylcyclase. J Biol Chem. 1991;266: 8595–8603.

48. Gallimore AR, Aricescu AR, Yuzaki M, Calinescu R. A Computational Model for the AMPA Receptor Phosphorylation Master Switch Regulating Cerebellar Long-Term Depression. Blackwell KT, editor. PLoS Comput Biol. Public Library of Science; 2016;12: e1004664. doi:10.1371/journal.pcbi.1004664

49. Bolia A, Gerek ZN, Keskin O, Banu Ozkan S, Dev KK. The binding affinities of proteins interacting with the PDZ domain of PICK1. Proteins. 2012;80: 1393–1408. doi:10.1002/prot.24034

50. Gianni S, Engström A, Larsson M, Calosci N, Malatesta F, Eklund L, et al. The kinetics of PDZ domain-ligand interactions and implications for the binding mechanism. J Biol Chem. American Society for Biochemistry and Molecular Biology; 2005;280: 34805–34812. doi:10.1074/jbc.M506017200

51. Czöndör K, Mondin M, Garcia M, Heine M, Frischknecht R, Choquet D, et al. Unified quantitative model of AMPA receptor trafficking at synapses. PNAS. 2012;109: 3522–3527. doi:10.1073/pnas.1109818109

52. Zawadzki KM, Taylor SS. cAMP-dependent protein kinase regulatory subunit type IIbeta: active site mutations define an isoform-specific network for allosteric signaling by cAMP. J Biol Chem. American Society for Biochemistry and Molecular Biology; 2004;279: 7029–7036. doi:10.1074/jbc.M310804200

53. Øgreid D, Døskeland SO. The kinetics of association of cyclic AMP to the two types of binding sites associated with protein kinase II from bovine myocardium. FEBS Lett. John Wiley & Sons, Ltd; 1981;129: 287–292. doi:10.1016/0014-5793(81)80185-8

54. Friedrich W Herberg, Susan S Taylor A, Dostmann WRG. Active Site Mutations Define the Pathway for the Cooperative Activation of cAMP-Dependent Protein Kinase†. Biochemistry. American Chemical Society; 1996;35: 2934–2942. doi:10.1021/bi951647c
